# Harp: data harmonization for computational tissue deconvolution across diverse transcriptomics platforms

**DOI:** 10.1093/bioinformatics/btaf455

**Published:** 2025-08-26

**Authors:** Zahra Nozari, Paul Hüttl, Jakob Simeth, Marian Schön, James A Hutchinson, Rainer Spang

**Affiliations:** Institute for Statistical Bioinformatics, Faculty of Informatics and Data Science, University of Regensburg, 93053 Regensburg, Germany; Institute for Statistical Bioinformatics, Faculty of Informatics and Data Science, University of Regensburg, 93053 Regensburg, Germany; Institute for Statistical Bioinformatics, Faculty of Informatics and Data Science, University of Regensburg, 93053 Regensburg, Germany; NGS and Data Technologies Core, Leibniz Institute for Immunotherapy (LIT), c/o Universitätsklinikum Regensburg, 93053 Regensburg, Germany; Institute for Statistical Bioinformatics, Faculty of Informatics and Data Science, University of Regensburg, 93053 Regensburg, Germany; Department of Surgery, Universitätsklinikum Regensburg, 93053 Regensburg, Germany; Institute for Statistical Bioinformatics, Faculty of Informatics and Data Science, University of Regensburg, 93053 Regensburg, Germany

## Abstract

**Motivation:**

The cellular composition of a solid tissue can be assessed either through the physical dissociation of the tissue followed by single-cell analysis techniques or by computational deconvolution of bulk gene expression profiles. However, both approaches are prone to significant biases. Tissue dissociation often results in disproportionate cell loss, while deconvolution is hindered by biological and technological inconsistencies between the datasets it relies on.

**Results:**

Using calibration datasets that include both experimentally measured and deconvolution-based cell compositions, we present a new method, Harp, which reconciles these approaches to produce more reliable deconvolution results in applications where only gene expression data is available. Both on simulated and real data, harmonizing cell reference profiles proved advantageous over competing state-of-the-art deconvolution tools, overcoming technological and biological batch effects.

**Availability and implementation:**

R package available at https://github.com/spang-lab/harp (archived as 10.5281/zenodo.16851930). Code and data for reproducing the results of this paper are available at https://github.com/spang-lab/harplication (archived as 10.5281/zenodo.16851705) and https://doi.org/10.5281/zenodo.15650057, respectively.

## 1 Introduction

Tissues consist of cells of different types. The relative frequencies of cells of specific types define the cellular composition of a tissue, which holds crucial information on its biology and pathology. It is altered in diseases such as cancers, chronic inflammations, or infections. While cell types can be coarsely distinguished by their shape, molecular data allows for a more finely granulated distinction of cells and even cell states. The more molecules considered, the better cells can be characterized.

Cellular composition can be assessed experimentally using single cell technologies such as fluorescence-activated cell sorting (FACS; Hu *et al.* 2016), cytometry by time-of-flight (CYTOF; Cheung and Utz 2011), single-cell RNA sequencing (scRNA-seq; Wu *et al.* 2014), or combinations of these methods. However, for solid tissues, a common limitation of these approaches is the bias introduced by enzymatic dissociation, which tends to disproportionately affect certain cell types, leading to their preferential loss during isolation (Wang *et al.* 2019, Denisenko *et al.* 2020, Kim *et al.* 2023).

An alternative approach is bulk gene expression profiling combined with computational deconvolution (Avila Cobos *et al.* 2018). In this method, a bulk expression profile is modeled as a weighted sum of reference profiles from individual cell types, where the weights represent the cellular composition of the tissue.

Let *X* be a g×q matrix representing reference profiles, where each column corresponds to a specific cell type and each row represents a gene. For the bulk data, let *Y* be a g×n matrix, where each column indicates a bulk profile and each row relates to a gene. Finally, for the cellular compositions, let *C* be a q×n matrix where every column is a bulk tissue and every row is a cell type. The entry $C_{ij}$ is the relative frequency of cell type *i* in tissue *j*. The central deconvolution equation connecting these data is


(1) Y=XC.

Building upon this equation, widely used tissue deconvolution tools including DTD (Görtler *et al.* 2020), CIBERSORTx (Newman *et al.* 2019), MuSiC (Wang *et al.* 2019), or ADTD (Görtler *et al.* 2024) estimate cellular abundances of the bulk samples. Furthermore, recent methods designed to estimate cell-type-specific gene expression, such as BayesPrism (Chu *et al.* 2022) and TissueResolver (Simeth *et al.* 2024), often provide remarkably accurate cellular composition estimates as a byproduct.

Deconvolution, comes with its own limitations (Garmire *et al.* 2024). In theory, Equation (1) should hold exactly. In reality, however, this equation does not hold, due to both tissue specific gene regulation and experimental inconsistencies in data generation. We distinguish two scenarios:

*Local inconsistencies:* Y=XC holds approximately for the majority of genes, but there is a small number of genes for which it is strongly violated. For example, if the references for T cells were generated from inactive T cells, while the bulk tissues contain activated T cells. In this case Equation (1) might hold for most genes, except for T cell activation markers. Experimental inconsistencies can also lead to this problem. For example, if a certain class of genes was experimentally depleted only in the bulk profiles but not the reference profiles. In this case, Equation (1) is mathematically infeasible for the depleted genes. Moreover, if reference profiles are derived from single-cell sequencing data, there can be substantial technological discrepancies compared to the bulk sequencing data used for tissues. Single-cell data is typically zero-inflated due to high drop-outs (Haque *et al.* 2017, Zheng *et al.* 2017), influenced by transcriptional burst (Chubb *et al.* 2006), and until recently, did not commonly include ribosomal RNA depletion (Shek *et al.* 2021), unlike bulk RNA sequencing.

*Global inconsistencies:* Y=XC does not hold for any of the genes, because there are global inconsistencies between the bulk and reference data. This situation typically occurs if different profiling technologies such as scRNA-seq and microarrays were used (Brombacher *et al.* 2025).

**Figure 1.**
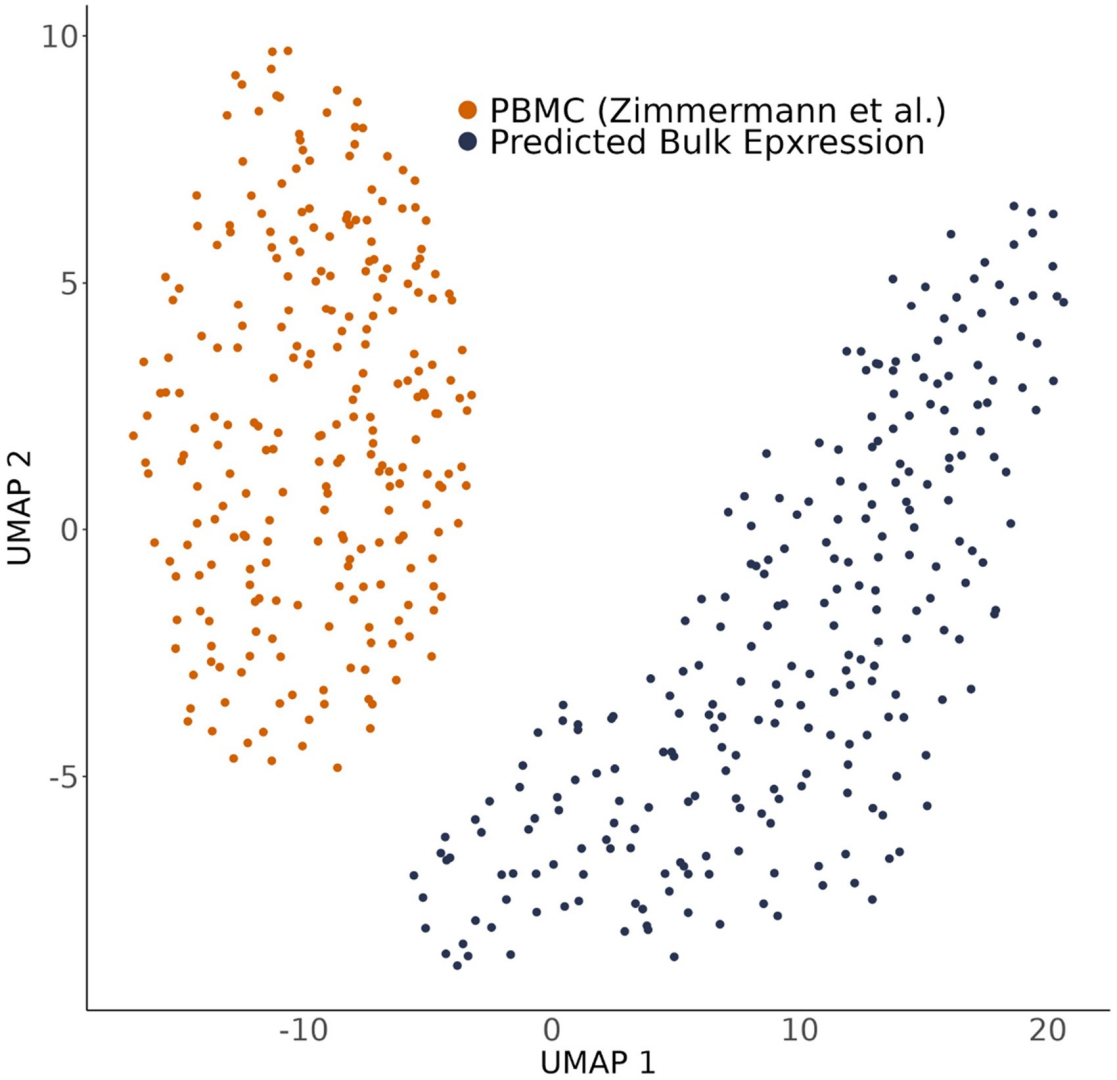
Comparison of bulk RNA-seq expression profiles to reconstructed bulk gene expression samples. Bulk RNA-seq data from (Zimmermann *et al.* 2016) is represented in orange, while reconstructed bulk expression, derived by combining flow cytometry data of the same bulk samples with cell type-specific signatures from sorted RNA-seq data (Monaco *et al.* 2019), is shown in navy.

Both local and global systematic differences prevent reference profiles from accurately summing up to bulk profiles. For example, Fig. 1 compares bulk RNA-seq data to a weighted average of sorted RNA-seq data, with the weights determined experimentally using flow cytometry. In the UMAP (Healy and Mclnnes, 2024) plot, the measured bulk profiles and the reconstructed profiles are clearly separated.

Several approaches to compensate for data inconsistencies have been applied and described in the literature. When local inconsistencies are known, affected genes can be manually excluded from the deconvolution, as in depletion protocols. If the affected genes are not known a priori, such as in tissue-specific gene regulation, machine learning-based approaches have been proposed to detect these inconsistencies from the data. For example, Digital Tissue Deconvolution (DTD) (Görtler *et al.* 2020) assumes that subsets of genes are affected by inconsistencies and eliminates those automatically from the deconvolution using a loss function learning approach. BayesPrism (Chu *et al.* 2022) in contrast, targets global inconsistencies involving all genes by marginalization of a posterior distribution conditioned on bulk and single cell expression data. CIBERSORTx provides two custom batch effect removal strategies. The first estimates an explained bulk expression matrix and then applies classical batch correction (Johnson *et al.* 2007) to adjust this estimation to the actual bulk expression, which is only possible for moderate batch effects. The second approach directly adjusts the signature matrix used for deconvolution by integrating single-cell information. There, artificial bulk mixtures are generated from single-cell data and then batch corrected, using again the method in (Johnson *et al.* 2007), in order to fit the actual bulk expression. Via non-negative least squares regression, taken into account the adjusted bulk mixtures and prior estimates of cellular frequencies, the adjusted signature matrix is then imputed. However, a method that systematically harmonizes possibly compromised cellular quantification measurements with transcriptomic data of various platforms is still lacking in the literature.

Here, we introduce Harp, a method that harmonizes reference profiles and measured cell compositions to improve the consistency and accuracy of computational tissue deconvolution. Harp addresses the limitations of existing approaches by explicitly integrating measured cellular compositions during training and aligning them with bulk expression data. This allows the method to correct for inconsistencies across transcriptomics platforms and reference sources. We demonstrate that Harp improves deconvolution performance in both simulated and real datasets, including challenging cross-platform scenarios, and outperforms existing methods in a range of evaluation metrics.

## 2 Materials and methods

### 2.1 Algorithm

**Figure 2.**
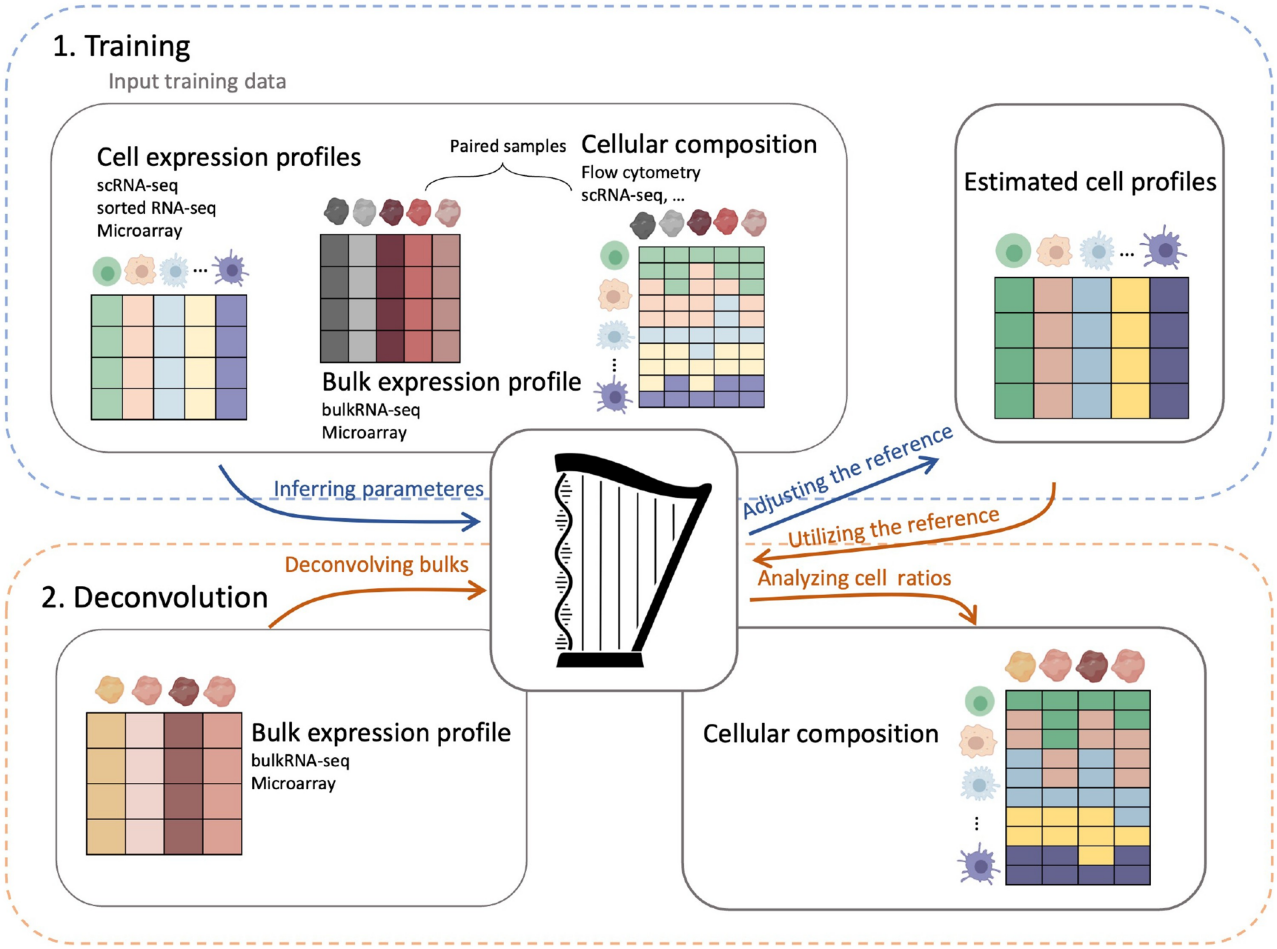
The Harp framework consists of two main modes: *Training* and *Deconvolution*. During *Training*, both bulk expression data and experimentally determined cell frequencies are used, whereas *Deconvolution* relies solely on expression profiles. In the *Training* step, Harp takes the following inputs: (i) a matrix of reference cell profiles derived from experiments on sorted cell populations using either RNA-seq or microarrays, or from single-cell RNA sequencing (scRNA-seq); (ii) bulk gene expression profiles obtained from either bulk RNA-seq or microarray technology; and (iii) a corresponding cellular composition matrix, generated using methods such as scRNA-seq, flow cytometry, or other techniques. Using these input data, Harp estimates a matrix of harmonized cell reference profiles. In the *Deconvolution* step, Harp takes new bulk gene expression samples, along with the estimated reference profiles from the *Training* step, to infer cellular compositions.

An overview of the Harp framework is provided in Fig. 2. Harp operates in two modes: *Training* and *Deconvolution*.

*Training mode:* In this mode, Harp takes as input a matrix *Y* of bulk tissue gene expression profiles, a corresponding matrix *C* of experimentally determined cellular tissue compositions, and a matrix of reference profiles *X*, holding cell signatures of these tissues (see also Section A.1, available as supplementary data at *Bioinformatics* online). These inputs may exhibit inconsistencies such that Y≠XC. The aim of Harp is to harmonize these inputs by adjusting both *X* and *C* to meet the following objectives:

- The adjusted cellular compositions, $C^{\prime }$, accurately represent the cellular composition of the tissue.
- The adjusted reference profiles, $X^{\prime }$, reflect the expression states of the cell types as they exist in the tissue.
- The relationship $Y\approx X^{\prime }C^{\prime }$ is approximately satisfied, where $X^{\prime }$ and $C^{\prime }$ are the harmonized versions of the input reference profiles and cellular compositions, respectively.

First, Harp accounts for potential errors in the composition matrix *C* by allowing some flexibility, facilitating data harmonization. In order to correct for these errors, it represents the cellular decomposition of the tissue by a parameterized matrix $C^{\prime }(\alpha )=\text{diag}(\alpha )C^{*}$, where


$$\begin{array}{c} C^{*}=\left(\begin{array}{c} C\\ C_{UI} \end{array}\right) \end{array}$$

is a (q+1)×n matrix that is identical to *C* but includes an additional row representing a mixture of all cell types that could be present in the tissue but are not accounted for in the cellular composition matrix (Racle *et al.* 2017, Görtler *et al.* 2024) (see Section A.2.3, available as supplementary data at *Bioinformatics* online for more details). The diagonal matrix diag(α), where α is a (q+1)-dimensional vector of non-negative scaling parameters accounts for cell-type-specific losses during the experimental determination of *C*. Then, since cell reference data often contains signatures from more cell types than those measured in flow cytometry data, *C*, we introduce an additional column to *X*, denoted as $X_{UI}$. This column represents the gene-wise average expression across all remaining cell types not captured in the flow cytometry data. We define this reference matrix as $X^{*}=(X,X_{UI})$.

Next, Harp adjusts the reference matrix $X^{*}$ to $X^{\prime }$ to meet two key criteria:

-
$Y\approx X^{\prime }C^{\prime }(\alpha ).$

- The columns of $X^{\prime }$ maintain similarity to the original anchor matrix $X^{*}$.

Criterion (b) is crucial to avoid artifacts caused by underdetermination, where $Y=X^{\prime }C^{\prime }(\alpha )$ may hold perfectly, but the adjusted profiles in $X^{\prime }$ no longer reflect the true biological expression patterns of the cell types they represent.

More formally, we minimize the following loss function with respect to $\phi^{*}$ and α simultaneously, where $\phi^{*}=(\phi ,\phi_{UI})$ with ϕ being a matrix of same dimension as *X* and $\phi_{UI}$ being an additional column accounting for the Unidentified cell types in compatibility with the extra row in $C^{*}$,


(2) $$L(\phi^{*},\alpha )={\left\|Y-\phi^{*}\text{diag}(\alpha )C^{*}\right\|}_{2}^{2}+\lambda R(X^{*},\phi^{*}),$$

with ${\left\|\cdot \right\|}_{2}$ denoting the Frobenius norm and *R* being defined as


(3) $$\begin{array}{c} R(X^{*},\phi^{*})=\sum_{ij}[\ln\left(1+\text{exp}({\left(\varphi -x\right)}_{ij})\right)+\\ \ln\left(1+\text{exp}({\left(-\varphi +x\right)}_{ij})\right)]. \end{array}$$

Note that the regularization term *R* constrains the adjusted references $X^{\prime }$ to remain close to the measured references. It anchors $X^{\prime }$.

Starting with diag(α) being equal to the identity matrix, the optimization process alternates between updates of $X^{\prime }$ using


(4) $$X^{\prime }={\text{argmin}}_{\phi^{*}}L(\phi^{*},\alpha )$$

followed by the update of α, by minimizing the loss function


(5) $$U(\alpha )={\left\|\text{diag}(\alpha )C^{*}-\hat{C}\right\|}_{2}^{2},$$

with respect to α, where $\hat{C}$ is the estimated cellular composition using $X^{\prime }$ as the reference profile, for more details see Section A.2.5, available as supplementary data at *Bioinformatics* online. Note that after $X^{\prime }$ is updated, its columns are normalized to sum up to the number of features (genes) it includes; this is necessary to keep both $X^{\prime }$ and α identifiable.

Additionally, we provided the option for Harp to automatically determine the regularization strength from a given range of λ values via a cross validation approach in order to arrive at an optimal $\lambda^{\prime }$ that balances both criteria (a) and (b), see Section A.2.2, available as supplementary data at *Bioinformatics* online.

*Deconvolution mode:* In this mode, Harp uses the adjusted reference profile matrix $X^{\prime }$, obtained during training, to deconvolve bulk tissue gene expression data sources similar to those used in training (e.g. comparable tissues or profiling technologies) where no experimentally determined tissue composition is available. By default, Harp applies the adjusted matrix $X^{\prime }$ in combination with the DTD algorithm (Görtler *et al.* 2020) for deconvolution in order to arrive at its final cell proportion estimate $C^{\prime }$, see also Section A.2.5, available as supplementary data at *Bioinformatics* online. However, the harmonized matrix $X^{\prime }$ can also serve as a reference profile for use with other deconvolution methods.

### 2.2 Performance metrics

Here, we introduce several established performance metrics to evaluate deconvolution results. They either compare cell abundance estimates to ground-truth cell proportions—which may either be predefined in simulation scenarios or obtained experimentally (e.g. through flow cytometry or scRNA-seq)—or predicted bulk gene expression to observed bulk expression data. For their mathematical definition we refer to Section A.2.4, available as supplementary data at *Bioinformatics* online.

*Cell type-specific performance:* The first performance metric of a deconvolution tool, $R_{c}(l)$, is its ability to accurately capture variations in the relative abundance of a given cell type *l* across different samples. For example, this quality metric can evaluate how accurately the method predicts that sample 1 contains 10% more T cells than sample 2.

*Sample-specific performance:* A second performance metric is the tool’s ability to accurately estimate the proportions of different cell types within an individual sample *m*, denoted as $R_{s}(m)$. For instance, $R_{s}$ can evaluate how accurately the method determines that one sample contains 30% more T cells than B cells.

*Combined performances:* Following (Wang *et al.* 2019) the cell type-specific and sample-specific performance metrics can be integrated into a single overall measure denoted as R. We furthermore include the absolute quality metrics RMSD and mAD into our analyses.

*Bulk reconstruction performance:* Thus far, our performance metrics have focused on evaluating the estimated cellular compositions *C*. In addition to accurately recovering these compositions, a well-calibrated deconvolution tool should also be able to recapitulate the observed bulk expression *Y* via the model


Y=XC.

To assess this, we introduce a bulk-centered performance measure ρ(m), which correlates explained bulk expression of sample *m* to its observed bulk expression.

*Statistical testing:* We evaluate the significance of performance improvements using a *z*-test on the correlation coefficients $R_{c}$ and $R_{s}$, following the approach of (Chu *et al.* 2022); see Section A.3.7, available as supplementary data at *Bioinformatics* online for details.

## 3 Results

### 3.1 Simulation

To evaluate the performance of Harp and compare it with state-of-the-art deconvolution algorithms, we performed extensive benchmarking simulations. In these simulations, we followed a well-established approach by generating artificial bulk expression profiles as weighted averages of single-cell expression profiles (Newman *et al.* 2019, Wang *et al.* 2019, Chu *et al.* 2022). Importantly, the simulated profiles allow us to control the “cellular composition” through the assignment of weights. For instance, in a profile where T cells constitute 30% of the cellular composition, the cumulative weight of the T cell profiles accounts for 30% of the total. In our simulations, we used single-cell data from two studies on non-Hodgkin lymphomas (nHL) (Roider *et al.* 2020, Steen *et al.* 2021). The study by Steen *et al.* (2021) comprises profiles from 28 416 single cells collected from eight patients, including four patients with Diffuse Large B-cell Lymphoma (DLBCL), three patients with Follicular Lymphoma (FL) and one control patient with Tonsilitis (T). The cells have been pre-annotated with the following cell type labels: B cells, Monocytes, Natural Killer cells, Plasmablasts, CD4 T cells, CD8+ T cells, regulatory T cells, T follicular helper cells, and a remaining unknown compartment. The second study, conducted by Roider *et al.* (2020), includes 35 284 single-cell profiles from 12 nHL cases, including three DLBCL, four FL, two Transformed Follicular Lymphoma (tFL) and three control patients exhibiting reactive lymph nodes (rLN). Similarly, these cells have been pre-annotated as B cells, myeloids, CD8+ T cells, regulatory T cells, follicular helper T cells, and T helper 1 cells. Notably within this study the B cells were further divided into healthy B cells and malignant lymphoma cells. For further details see Supplement, Section A.3.2, available as supplementary data at *Bioinformatics* online and Fig. 9, available as supplementary data at *Bioinformatics* online.

We observed substantial inconsistencies and batch effects between these studies (also see Fig. 7a, available as supplementary data at *Bioinformatics* online), which can be partially attributed to differences in laboratory protocols, tissue handling, and library preparation, as well as patient heterogeneity (see Supplementary Material, Section A.3.1, available as supplementary data at *Bioinformatics* online). We used these discrepancies to simulate inconsistent datasets. Specifically, the data from the first study defined the reference profiles, while the data from the second study were used to generate artificial bulk samples. For Harp’s anchor $X^{*}$ we used the average cell-type specific expression across single cell profiles of (Steen *et al.* 2021). Concerning (Roider *et al.* 2020), we randomly divided the data from 12 patients into two independent sets of six patients each. One set was used to generate a training set of artificial bulk samples, while the other set was used to create an independent test set. For deconvolution we focused on only those cell types that were defined in both studies, namely B cells, CD8+ T cells, regulatory T cells and follicular helper T cells. Importantly, the B cell compartment contained both malignant and physiological cells from this B cell malignancy. This implies completeness of the reference and thus, $X^{*}=X$.

For each bulk sample, we randomly selected cells stemming from a single patient only. More precisely, we determined the actual amount of single cells for each cell type within a given patient and then perturbed this amount with a normally distributed factor in order to arrive at the quantity of cells to be randomly selected from each cell type. This allowed us to generate multiple artificial bulk mixtures from a single patient, which contain suitable variation in terms of cellular composition (see Section A.3.3, available as supplementary data at *Bioinformatics* online). Following (Chu *et al.* 2022), we introduced additional distortions that further amplify the discrepancies observed between the datasets, by locally perturbing gene expression values in artificial bulk mixtures with a gene specific multiplicative noise. More precisely, we sampled gene-specific factors from a pre-defined normal distribution and then multiplied this factor to the expression value of the considered gene in all artificial bulk mixtures. Following this approach, we perturbed 40% of all genes while the remaining set of genes was left unchanged (see Section A.3.4, available as supplementary data at *Bioinformatics* online). In total, we generated 20 artificial training samples and 40 test samples using this protocol (also see Supplement, Section A.3.5, available as supplementary data at *Bioinformatics* online). Figure 11, available as supplementary data at *Bioinformatics* online shows the distribution of cell proportions in these datasets. Importantly, this simulation framework naturally controls the proportions of the various cell compartments in the artificial bulk mixtures, see Section A.3.3, available as supplementary data at *Bioinformatics* online for details.

#### 3.1.1 Calibration of regularization

Our first analysis addresses the calibration of the parameter λ in Equation (2). Regularization enforces a degree of similarity between the adjusted reference matrix $X^{\prime }$ and its unadjusted counterpart $X^{*}$. Note that overly strong regularization results in minimal adjustment of the reference profiles, potentially failing to compensate for technical discrepancies. On the other hand, weak or absent regularization can produce reference profiles that diverge from the true expression characteristics of the cells they represent. For example, the column corresponding to B cells in $X^{\prime }$ might no longer capture the typical expression profile of a B cell, indicating that the reference has been “over-adjusted.”

**Figure 3.**
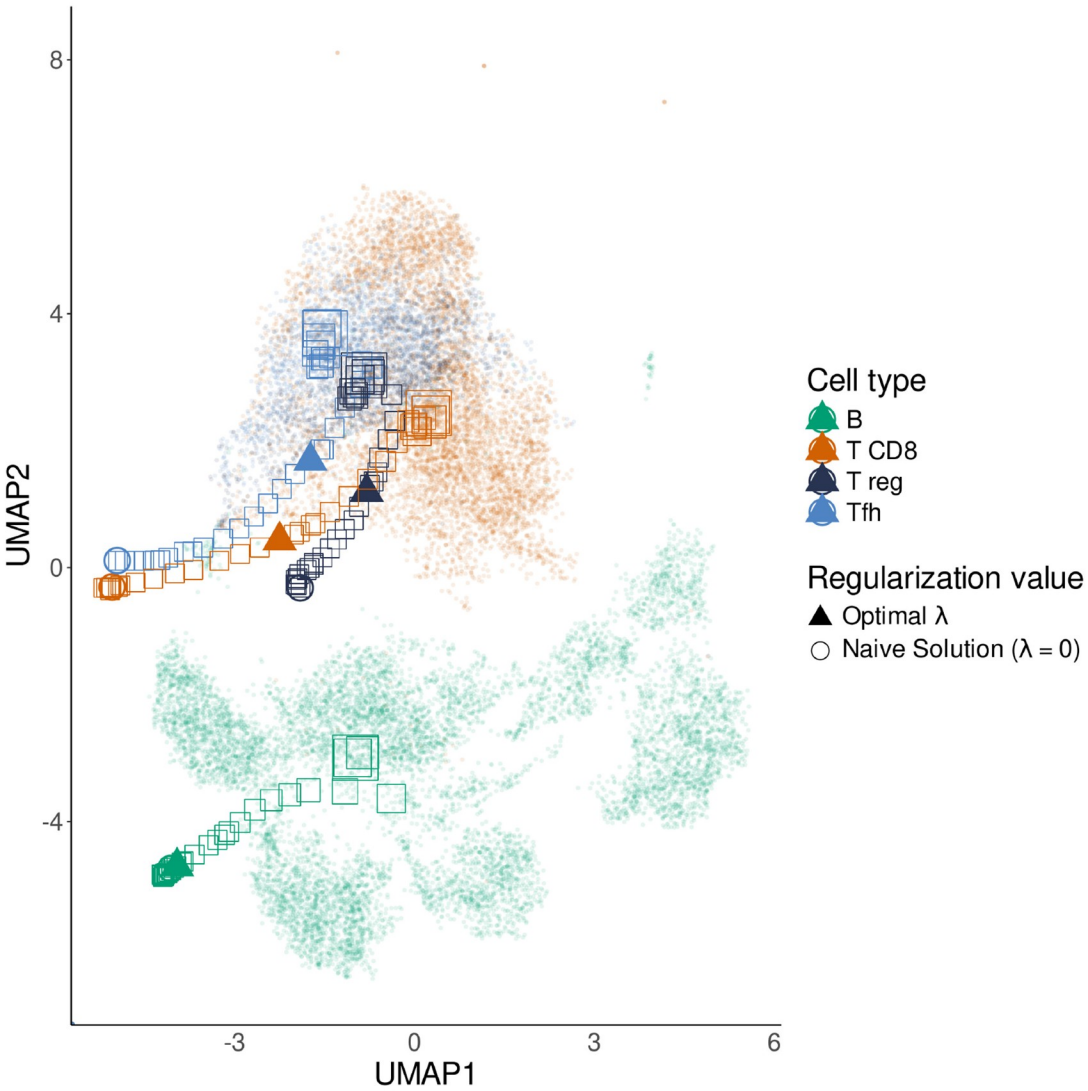
Reference profiles learned by Harp for different regularization parameter (λ) values, embedded in the single-cell context using UMAP. Each square represents a cell type-specific expression profile in the Harp reference for a given λ, with square size encoding the magnitude of λ. Triangles indicate the optimal estimated reference profiles selected by Harp, and each color corresponds to a specific cell type.

In order to better understand Harp’s dependency on its hyperparameter λ, we fitted models on simulated data using different λ values in the range $\left[0,2^{15}\right]$. Let $X^{\prime }(\lambda )$ be the adjusted reference matrix produced by Harp when using regularization strength λ. Figure 3 shows a UMAP (Healy and Mclnnes 2024) embedding of (a) the single-cell profiles used in the training data and (b) the columns of $X^{\prime }(\lambda )$ containing the adjusted reference profiles for various cell types at different values of λ. In the plot, small dots represent single-cell expression profiles, with colors indicating their corresponding cell types. In contrast, squares denote reference profiles extracted from $X^{\prime }(\lambda )$, where the square size increases with larger λ values. Triangles represent the reference profiles obtained for the optimal $\lambda^{\prime }$, which are the adjusted references used by Harp in *Deconvolution* mode, see Section 2.1.

We observed that large λ values yield reference profiles located at the centers of the corresponding single-cell clusters, indicating minimal adjustment. As λ decreases, adjustments become visible as the reference profiles gradually shift away from the cluster centers; however, except for very small λ values, they remain in the vicinity of the clusters they represent. Optimal reference profiles tend to lie along this trajectory toward the center, reflecting the typical bias-variance trade-off observed in machine learning applications.

#### 3.1.2 Benchmarking

**Figure 4.**
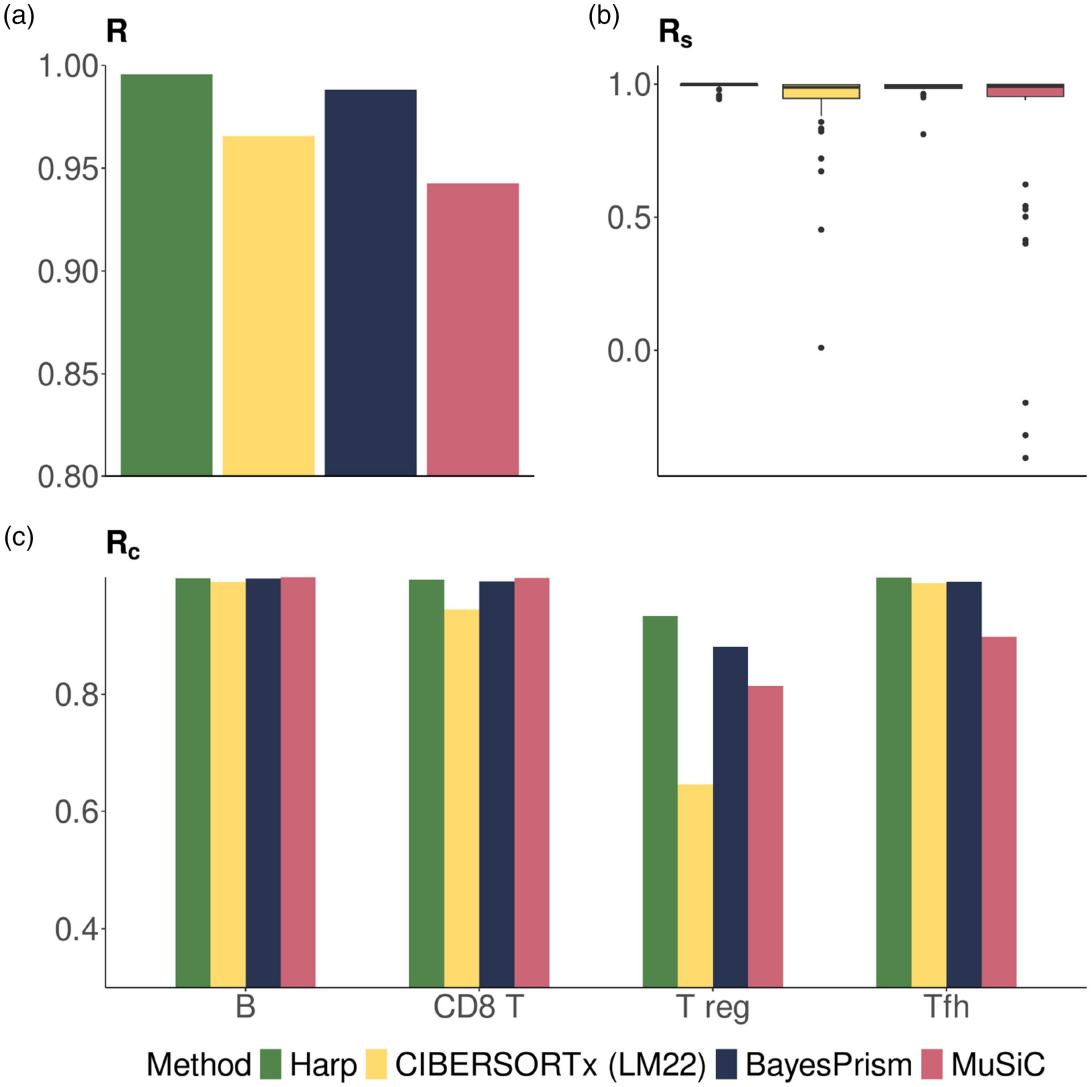
Evaluation of performance metrics in simulated data. (a) shows the overall correlation performance, R, (b) the sample-specific performance, $R_{s}$, and (c) cell type-specific performance, $R_{c}$.

We benchmarked Harp’s performance against a set of widely used deconvolution tools, including BayesPrism (Chu *et al.* 2022), CIBERSORT (Newman *et al.* 2015), CIBERSORTx (Newman *et al.* 2019), and MuSiC (Wang *et al.* 2019). Harp was trained on the training set of bulk mixtures with known ground truth proportions and subsequently evaluated in *Deconvolution* mode on the independent test dataset. Since none of the other algorithms incorporate a training phase for data harmonization, they were applied directly to the bulk samples in the test data, see Section A.3.6, available as supplementary data at *Bioinformatics* online for details. Figure 4, Fig. 8, available as supplementary data at *Bioinformatics* online, and Tables 1 and 2, available as supplementary data at *Bioinformatics* online demonstrate that in these simulations, Harp significantly outperformed its competitors across all performance metrics introduced in Section 2.2.

One might argue that, in the previous benchmark, Harp had an advantage, because it was trained with additional data, including ground-truth compositions, and could perform data harmonization—a capability not available to the competing algorithms. To test whether harmonization could also improve the performance of these methods, we provided the harmonized reference matrix, $X^{\prime }$, calculated by Harp to competing deconvolution tools, see Sections A.3.6 and A.3.9, available as supplementary data at *Bioinformatics* online for details. Figure 8, available as supplementary data at *Bioinformatics* online and Tables 1 and 2, available as supplementary data at *Bioinformatics* online show that using Harp’s reference in this *hybrid* deconvolution scenario is highly beneficial when provided to CIBERSORTx, in comparison to the default use of BayesPrism as well as CIBERSORTx.

#### 3.1.3 Uncertain experimental compositions

Previous evaluations assumed that the composition matrix *C* was fixed by simulation design and served as ground truth. In real applications, however, *C* is experimentally determined and may suffer from both systematic bias and random noise. Harp addresses systematic bias through cell type-specific correction rates α, which are integrated into the loss function [see Equation (2)]. We next evaluate the effectiveness of this correction mechanism.

To this end, we simulated training and test bulk mixtures as before, yielding ground truth proportions C(l) by design. We then introduced systematic bias by multiplying each cell type’s proportion with a fixed, cell type-specific distortion factor δ(l), simulating effects such as cell loss or consistent gating errors. This distortion was constant across all samples in the training set (see Section A.3.10, available as supplementary data at *Bioinformatics* online).

To model random noise, we further multiplied the ground truth proportions with sample- and cell-type-specific random distortions. This step reflects the variability often seen in experimental quantification of cell type proportions.

We then ran Harp in *Training* mode twice: once using the correct cell proportions and once with the distorted proportions. Figure 12, available as supplementary data at *Bioinformatics* online and Tables 1 and 2, available as supplementary data at *Bioinformatics* online demonstrate that cell type-spcific distortions had minimal impact on Harp’s overall performance. Moreover, examining the estimated parameters α(l) alongside the distortion rates δ(l) reveals that, as expected, $\alpha (l)\approx \delta {\left(l\right)}^{-1}$, see Fig. 13, available as supplementary data at *Bioinformatics* online.

### 3.2 Data harmonization with Harp improved deconvolution accuracy in a study combining data from two distinct sources

We assessed Harp’s performance by integrating data from two sources. The bulk RNA-seq data were obtained from a study investigating primary peripheral blood mononuclear cells (PBMCs) in healthy individuals following influenza vaccination (Zimmermann *et al.* 2016). We utilized bulk RNA-seq expression profiles for 250 cases, with the cellular composition of the PBMCs experimentally determined via flow cytometry. However, this study did not include reference profiles for the various PBMC cell types. For the cell expression references we used data from an independent source (Monaco *et al.* 2019), which generated RNA-seq profiles of sorted PBMC cell populations. Details on data preprocessing can be found in Section A.4.1, available as supplementary data at *Bioinformatics* online.

**Figure 5.**
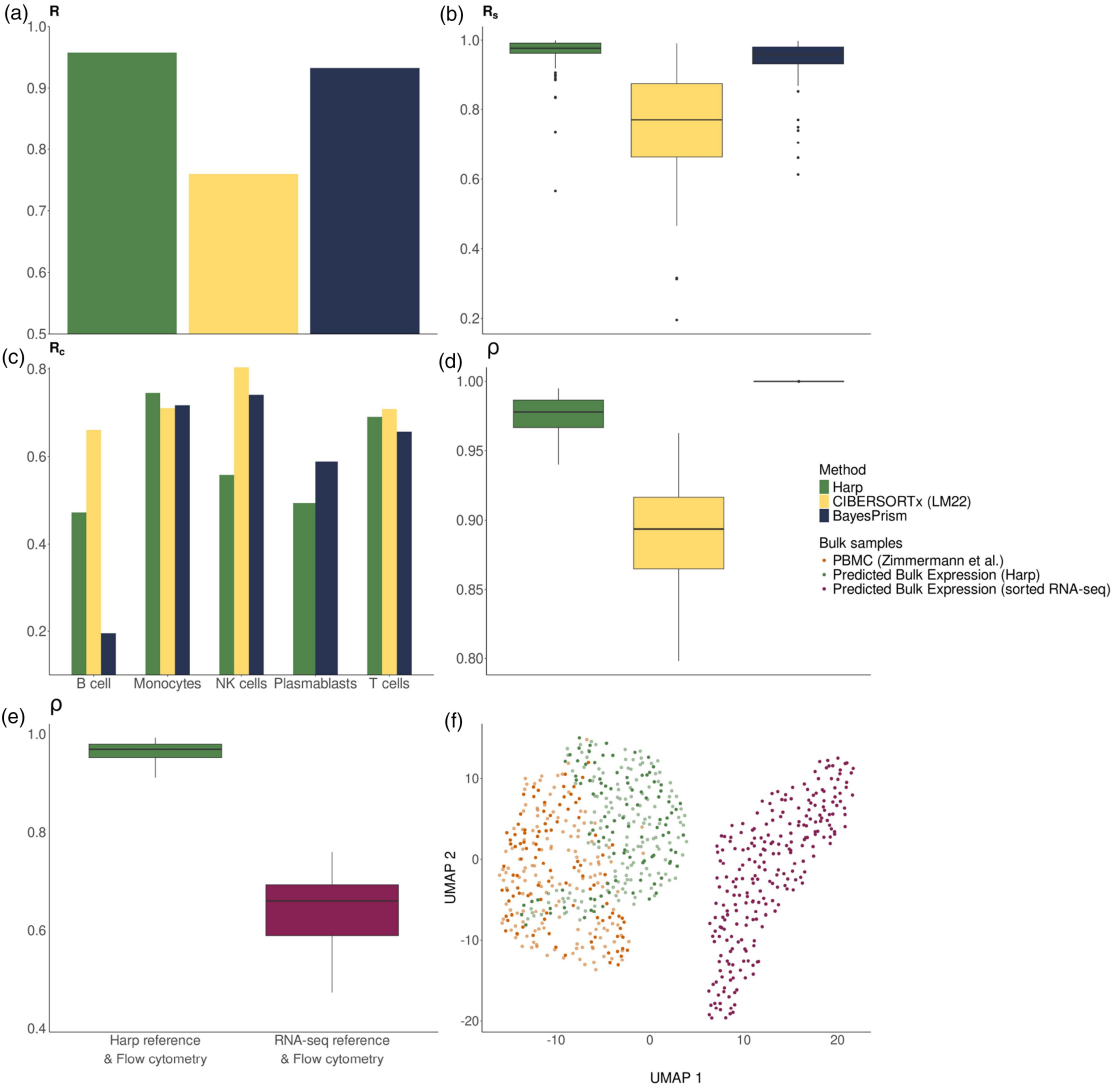
Evaluation of performance metrics in the deconvolution of 100 PBMC RNA-seq test samples with a sorted RNA-seq reference. (a–d) represent the benchmark of deconvolution tools [Harp, CIBERSORTx (LM22), and BayesPrism]. (a–c) evaluate performance on the prediction of cell proportions, while plot (d) analyses the quality of the reconstructed bulk gene expression profiles. (e) shows box plots of Pearson correlations, ρ, between the reconstructed bulk gene expression profiles—generated using the experimental cellular compositions in conjunction with the Harp (green) and sorted RNA-seq derived (magenta) references, respectively—and the observed bulk RNA-seq data. (f) is a UMAP of the predicted bulk gene expressions of 100 PBMC test samples (darker color shades) and 150 PBMC training samples (represented in lighter color shades), using the Harp (green) and sorted RNA-seq (magenta) reference, respectively, in conjunction with the cellular compositions derived from experimental data. This plot also includes observed bulk RNA-seq expression profiles (orange).

We randomly split the bulk data into a training set of 150 cases and a test set of 100 cases. For the training set, we ran Harp using the reference data from the second source as the anchor $X^{*}$ for regularizing the reference profile. We then applied Harp in *Deconvolution* mode to the test bulk samples, alongside CIBERSORTx and BayesPrism, and compared the deconvolution results to the corresponding flow cytometry measurements (see details on the configurations of other algorithms in Section A.4.2, available as supplementary data at *Bioinformatics* online). Figure 5a–c and Table 3, available as supplementary data at *Bioinformatics* online show that Harp outperformed its competitors in several, though not all, performance metrics. Most notably, it achieved robust overall performance, as indicated by the metric R (also see RMSD and mAD in Fig. 17, available as supplementary data at *Bioinformatics* online and Table 3, available as supplementary data at *Bioinformatics* online), which was supported by excellent sample-specific reconstructions of cell proportions. In statistical testing, Harp showed significant improvement in terms of sample-specific performance but not in cell type-specific correlation, compared to the competing methods (for details, refer to Section A.3.8, available as supplementary data at *Bioinformatics* online and Table 4, available as supplementary data at *Bioinformatics* online). In addition to the evaluation of cell proportion reconstruction, we evaluated data consistency after harmonization by reconstructing bulk expression profiles *Y* using reference data *X* and cell compositions *C* according to the formula Y=XC. We performed this reconstruction twice: once using the original (anchor) reference $X^{*}$ and once using the harmonized reference $X^{\prime }$ estimated by Harp. The cell abundances, *C*, used in both sets of reconstructed bulk samples were obtained from flow cytometry data (also see Section A.4.3, available as supplementary data at *Bioinformatics* online). Figure 5e shows that the bulk reconstructions based on Harp’s reference exhibited a higher correlation with the observed bulk profiles. This improved consistency was even more evident when both observed and reconstructed bulk profiles were embedded in a UMAP, see Fig. 5f.

We also compared the performance of the methods in terms of the reconstruction of bulk samples using each method’s corresponding reference and estimated cell proportions (see details in Sections A.4.2 and A.3.8, available as supplementary data at *Bioinformatics* online). As shown in Fig. 5d and Table 3, available as supplementary data at *Bioinformatics* online, Harp performed better than CIBERSORTx (LM22), while BayesPrism achieved the best performance. However, in regard of this quality metric BayesPrism always showed the strongest correlation (≈1) independent of the provided dataset. This is likely due to an explicit constraint in the method’s optimization approach, which forces reconstructed expressions to match the original data values (Chu *et al.* 2022).

Similarly to the simulation experiments, we provided the harmonized reference matrix learned by Harp to CIBERSORTx and BayesPrism, and compared the performance of this approach, to that achieved with their respective references.

Figure 17, available as supplementary data at *Bioinformatics* online and Table 3, available as supplementary data at *Bioinformatics* online show clear gains in overall and sample-wise performance, as well as bulk reconstruction ability when using Harp’s reference compared to a method’s default reference, though cell type-specific performance yielded mixed results (see Sections A.4.4 and A.4.2, available as supplementary data at *Bioinformatics* online for additional details and Section A.3.8, available as supplementary data at *Bioinformatics* online for the statistical analysis).

**Figure 6.**
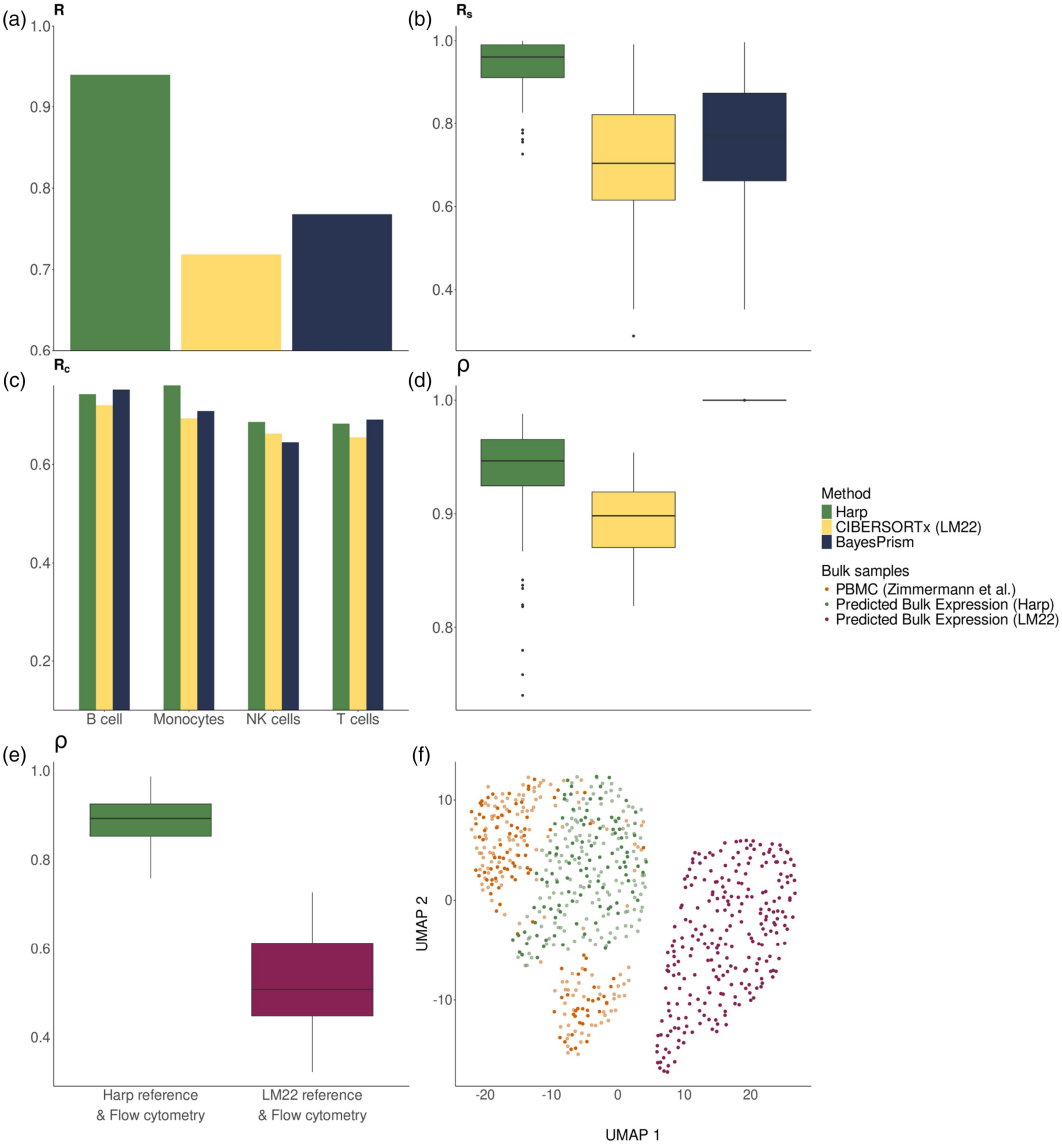
Evaluation of performance metrics in the deconvolution of 100 PBMC RNA-seq test samples with a microarray-based reference (LM22). (a–d) represent the benchmark of various deconvolution tools [Harp, CIBERSORTx (LM22), BayesPrism]. (a–c) evaluate performance in predicting cell proportions, while Plot (d) analyses the quality of the reconstructed bulk gene expression profiles. (e) shows box plots of the Pearson correlation, ρ, between the reconstructed bulk gene expression profiles, using the flow cytometry-derived cell proportions, together with the Harp (green) and LM22 (magenta) references. (f) is a UMAP of the predicted bulk gene expressions of 100 PBMC test samples (darker color shades) and 150 PBMC training samples (represented in lighter color shades), using the Harp (green) and a microarray-based (magenta) reference (LM22), respectively, in conjunction with the cellular compositions derived from experimental data. This plot also includes observed bulk RNA-seq expression profiles (orange).

So far we used data from different sources but comparable technologies, as both the bulk profiles and the anchor reference samples were derived using standard RNA-seq protocols. Next, we challenged Harp and its competitors further by using microarray-derived reference profiles as a starting point (anchor $X^{*}$) for harmonization. Our analysis is identical to that described above, with the sole difference that CIBERSORTx’s LM22 matrix, which is microarray-derived, replaces the references derived from RNA-seq profiles of sorted cell compartments (for details, see Section A.4.2, available as supplementary data at *Bioinformatics* online). Figure 6a–c and Table 6, available as supplementary data at *Bioinformatics* online show that, for cell proportion predictions, Harp outperformed both methods across all performance metrics (also see the results for RMSD and mAD in Fig. 18, available as supplementary data at *Bioinformatics* online), except for $R_{c}$ in the B cell compartment. Nonetheless, the performance of all methods with respect to the $R_{c}$ metric was comparable for most cell types. Statistical testing also showed that Harp significantly performed better than other methods in sample-specific performance but did not present a siginigicant improvement in cell type-specific performance (for details see Section A.3.8, available as supplementary data at *Bioinformatics* online and Table 7, available as supplementary data at *Bioinformatics* online). Figure 6d shows that both Harp and BayesPrism, when using LM22, explained the bulk gene expression samples better than CIBERSORTx.

Moreover, as shown in Fig. 6e, the advantage of using the Harp estimated reference over the microarray-based LM22 reference for bulk reconstruction was pronounced, with the average correlation improving from approximately 0.5 to about 0.9 (see also Section A.4.3, available as supplementary data at *Bioinformatics* online). Embedding both the observed and reconstructed bulk profiles into a UMAP further highlighted the improved consistency (Fig. 6f).

Finally, we again examined the effect of using harmonized references on the performance of competing tools. Figure 18, available as supplementary data at *Bioinformatics* online and Table 6, available as supplementary data at *Bioinformatics* online show that both CIBERSORTx and BayesPrism generally benefited from using Harp’s reference. Notably, when CIBERSORTx was used with Harp’s reference, no batch correction was performed when applying CIBERSORTx, yet its performance still improved (see the details in Sections A.4.2 and A.4.5, available as supplementary data at *Bioinformatics* online).

The benchmark comparison of deconvolution tools and *hybrid* deconvolution on microarray bulk expression data, where technological inconsistency is insignificant, is discussed in Section A.5, available as supplementary data at *Bioinformatics* online. Therefore, in this case, harmonization was not particularly required. The results indicate that Harp’s performance is comparable to its performance discussed earlier.

## 4 Discussion

We introduced Harp, a novel deconvolution tool designed for applications where reference data and bulk data are derived from different sources and are therefore not fully compatible. By performing data harmonization, Harp overcomes these discrepancies, emerging as a cross-platform deconvolution tool that enables analyses beyond the confines of a single data source.

Harmonization strongly depends on the technological platforms used for tissue processing, measurement of cellular composition, and gene expression profiling, as well as on the types and states of the tissues. In many cases, suitable training data are not publicly available and must be generated prior to deconvolution. Based on our experience, a small dataset of 20 bulk expression profiles with corresponding composition measurements provides a practical starting point.

A known challenge in tissue deconvolution is that cell types vary in RNA content (Monaco *et al.* 2019), which can lead to underestimation of those with lower RNA abundance. Harp addresses this by incorporating experimentally measured cellular compositions during training. When RNA yield differences are consistent across tissues, Harp’s scaling factor α [see Equation (5)] helps align RNA-based and experimental proportions. While α may also reflect technical variation—such as cell loss or protocol-specific biases—it provides a flexible mechanism to account for such systematic discrepancies.

We emphasize that in our work harmonization is not an end in itself; the ultimate goal is to accurately predict the cellular composition of a tissue. In its *Training* mode, Harp uses experimentally determined proportions of various cell compartments, adapting the reference profiles to achieve better overall consistency between these proportions, the reference data, and the bulk expression. However, the input cell compositions may be compromised by cell loss during tissue preparation, the omission of cell compartments that were present in the tissue, or errors during gating (manual or automated). As a result, pushing deconvolution results closer to these potentially flawed experimental measurements—as Harp does in *Training* mode—might be counterproductive, even if the deconvolution outcomes appear to better match the experimental data, as observed in our evaluations.

Moreover, deconvolution tools should not be seen merely as a way to replicate flow cytometry analyses. It is possible that computational deconvolution, in some cases, could yield more accurate estimates than experimental quantifications, as it accounts for signals from all cell compartments within the tissue—potentially capturing components that might otherwise be overlooked.

However, this leaves us with the challenge of determining which approach is more accurate, as a definitive ground truth does not currently exist (Garmire *et al.* 2024). We anticipate that this will change as both experimental protocols and image-based analyses progress rapidly. In the meantime, we advocate harmonizing all available information so that apparent discrepancies (e.g. those shown in Fig. 1) are addressed. Harp is designed to achieve precisely this.

## Supplementary Material

btaf455_Supplementary_Data

## Data Availability

The data supporting this article are available under GEO accession numbers GSE182436 and GSE182434 (single-cell data from Steen *et al.* (2021)), GSE107011 (data from Monaco *et al.* (2019)), and GSE65133 (data from Newman *et al.* (2015)), under doi:10.11588/data/VRJUNV (data from Roider *et al.* (2020)), and under SDY67 on https://science.bostongene.com/kassandra/downloads (processed data from Zimmermann *et al.* (2016)). Processed data are available on zenodo under doi:10.5281/zenodo.15650057 and doi:10.5281/zenodo.10139153.
