# Supplementary material for: Harp: data harmonization for computational tissue deconvolution across diverse transcriptomics platforms
Source: Bioinformatics. 2025 Aug 26;41(9):btaf455. doi: 10.1093/bioinformatics/btaf455 (PMC12448907; doi:10.1093/bioinformatics/btaf455)
Supplement: btaf455_Supplementary_Data [file btaf455_supplementary_data.pdf]

Supplemental Data

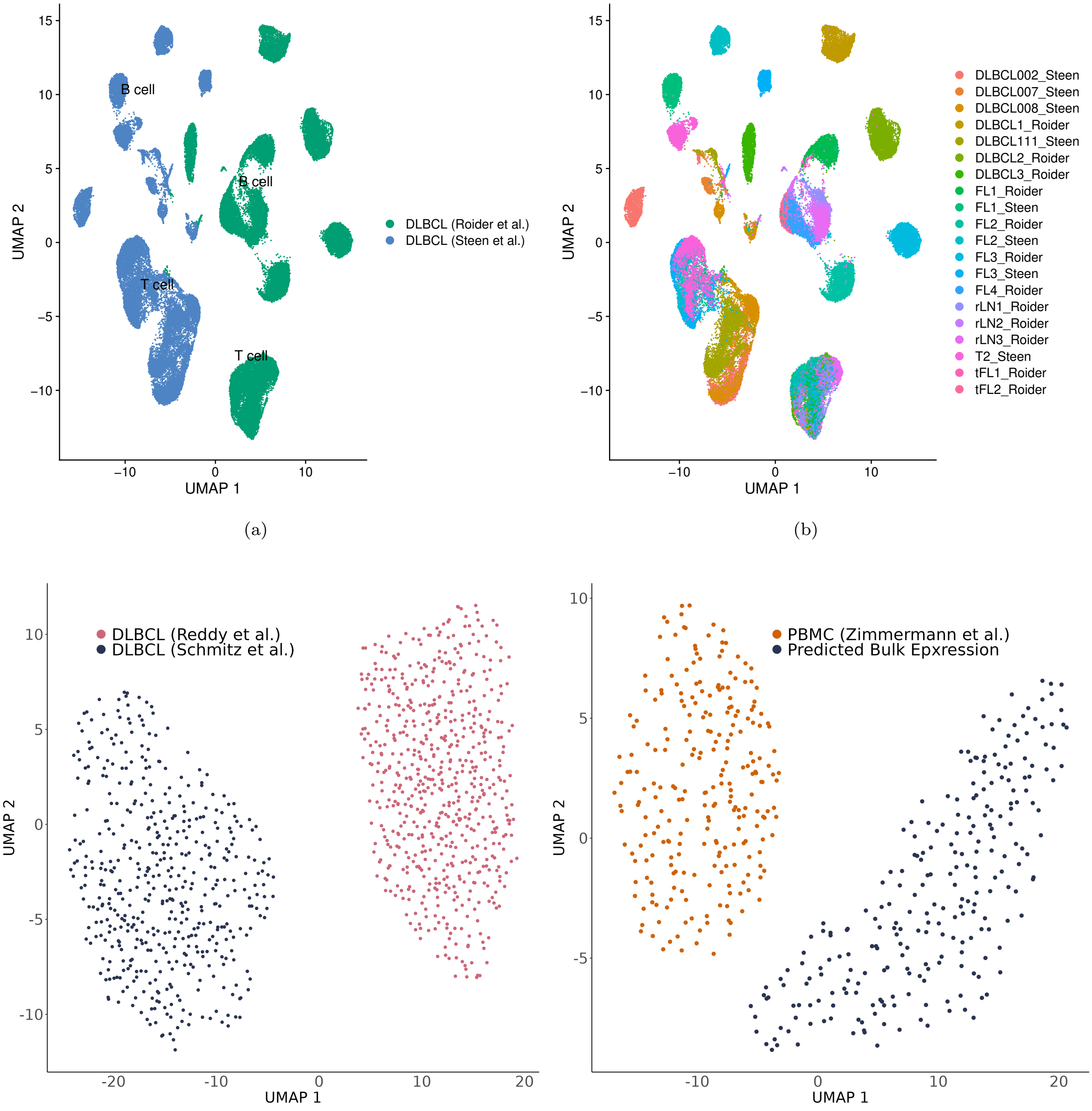

Figure. 7. (a) DLBCL single-cell profiles from two distinct studies: green represents data from (Roider et al. 2020), while blue corresponds to (Steen et al. 2021). (b) Same data as in (a), but colored by patient sample from the respective study. (c) DLBCL bulk RNA-seq expression data from two studies: pink refers to data from (Reddy et al. 2017), and dark blue illustrates (Schmitz et al. 2018) data. (d) Bulk RNA-seq expression of samples from (Zimmermann et al. 2016) is represented in orange, while reconstructed bulk expression—derived by multiplying flow cytometry data of the same bulk samples with cell signatures from sorted RNA-seq data (Monaco et al. 2019)— is shown in navy.

Appendix

A.1 Harp's input data

In the *Training* mode of Harp, the required input consists of three data types:

1. The cell reference profile, where each column represents the average gene expression for a specific cell type. This profile can be constructed using data from sorted bulk experiments (such as microarray or RNA-seq) or from scRNA-seq technologies.

2. The bulk gene expression matrix, which contains gene expression measurements with genes as rows and samples as columns. These measurements are typically obtained using RNA-seq or microarray technology.
3. The cellular composition matrix, which provides the known proportions of different cell types in each bulk sample described in item 2. This data can be obtained from single-cell technologies such as flow cytometry or scRNA-seq and must be derived from the same samples used to generate the bulk gene expression data.

We suggest at least 20 training samples when there are four to five cell types. If there are more cell types, a procedure similar to the one we used in our simulations with single-cell data should be followed to determine the optimal number of training samples.

In the *Deconvolution* mode, Harp requires bulk gene expression samples with unknown cellular compositions, which need to be inferred. Ideally, these samples should be measured using the same technology and originate from the same tissue context as the bulk data used during training.

The bulk gene expression data and cell type-specific reference profiles may come from different platforms or measurement technologies. Harp is specifically designed to address these discrepancies.

A.2 Method

A.2.1 Optimization algorithm

We numerically solved the optimization problem described in Section 2.1 via a custom gradient descent approach. We chose the anchor reference profile X^* as initial value for ϕ^* in the gradient descent. This is motivated by the fact that the final optimal reference profile X' shall encode biological information by perturbing X^* along the optimization process.

The partial derivative of the loss function, see equation (2), can be written as

$$\frac{\partial L(\phi^*, \boldsymbol{\alpha})}{\partial \varphi_{kl}} = -2\left[\sum_{m=1}^{n} y_{km}\alpha_l c^*_{lm} - \sum_{m=1}^{n}\sum_{p=1}^{q} \varphi_{kp}\alpha_p c^*_{pm}\alpha_l c^*_{lm}\right] + \lambda\left[(1+\exp((-\varphi+x)_{kl}))^{-1} - (1+\exp((\varphi-x)_{kl}))^{-1}\right], \tag{6}$$

where φ_{kl} is the expression level of gene k in cell type l. Using this gradient, we evolve ϕ^* via gradient descent

$$\phi^*_i = \phi^*_{i-1} - \eta \nabla L(\phi^*_{i-1}),$$

where $\eta > 0$ denotes the learning rate.

In order to automatically determine an optimal learning rate in each step, especially for balancing runtime efficiency and accuracy, we used Armijo backtracking, see (Nocedal and Wright 1999). More precisely in each gradient descent step the learning rate is iteratively decayed with the factor γ^k where $\gamma \in (0,1)$ and $k = 0, 1, 2 \ldots$ until the sufficient decrease condition

$$L(\phi^*_{i-1} - \gamma^k \eta \nabla L(\phi^*_{i-1})) - L(\phi^*_{i-1}) \leq -c_1 \gamma^k \|\nabla L(\phi^*_{i-1})\|_2^2,$$

is met. In our applications we chose $c_1 = 10^{-4}$ and $\gamma = 0.5$.

As convergence criterion we required smallness of the gradient, i.e., we stopped when

$$\left\|\nabla L(\phi^*_i)\right\|_1 < \varepsilon g n, \tag{7}$$

where we chose the hyperparameter $\varepsilon = 10^{-4}$. Due to the dimensionality of the problem we included both the number of genes, g, and the number of samples, n, in the breaking condition. However, in order to guarantee robust breaking in the case of non-convergence we limited the number of iterations to 10^5 and the minimal η during backtracking to 10^{-7}. We note, however, that in our applications, we always satisfied condition (7).

A.2.2 Regularization approach

In the loss function R (see equation (2)) serves as a regularization term controlled via the parameter λ. This regularization is essential to ensure the biological integrity of the reference profiles. Practically, it ensures the incorporation of single-cell information into the derived reference profile for $\lambda > 0$.

The user can either provide a desired regularization value directly or Harp can determine an optimal regularization value λ' via the following cross-validation approach. First, Harp uses a range of candidate λ values, which by default is given as the sequence

$$0, 0.1, 0.2, \ldots, 1, 2, 4, 8, 16, 32.$$

Next the provided training data set is split into n folds, with $n = 5$ by default. Now for each regularization value in the provided range, Harp is executed in *Training* mode with the exploited samples being comprised of $n-1$ folds, yielding a reference $X'(\lambda)$. With Y being given as the bulk samples from the remaining fold, we solve

$$\tilde{C}(X'(\lambda)) = \text{argmin}_\Gamma \left\|Y - X'(\lambda)\Gamma\right\|_2^2.$$

The solution is given as

$$\tilde{C}(\lambda) = (X'^\top(\lambda)X'(\lambda))^{-1}X'^\top(\lambda)Y. \tag{8}$$

In the next step, for each cell type we calculate the correlation, R_c, between these predicted cell abundances, $\tilde{C}(\lambda)$, and the cell proportions from the experiments. In cross-validation, this provides us with a cell type-specific quality score for all samples in the original training set. Thus, we consider the mean correlation across all cell-types and samples in order to arrive at a single score for each candidate λ value. The λ which provides the highest score is then selected as optimal λ' implying the use of $X'(\lambda')$ for the subsequent steps of the algorithm.

The regularization term R in equation (3) is given via the SoftPlus function which serves the purpose of being analytic at zero while being a suitable approximation of $\|\phi^* - X^*\|_1$. The scalar SoftPlus function is defined as

$$f(x) = \ln(1 + \exp(x)),$$

and its derivative is represented by

$$f'(x) = (1 + \exp(-x))^{-1}.$$

Therefore,

$$f(x) + f(-x) = \ln(1 + \exp(x)) + \ln(1 + \exp(-x)),$$

which can be approximated as

$$f(x) + f(-x) \approx \begin{cases} x & \text{if } x \gg 0 \\ -x & \text{if } x \ll 0 \\ 2\ln(2) & \text{if } x = 0 \end{cases}.$$

Thus, the absolute value function can be approximated as

$$|x| \approx f(x) + f(-x).$$

A.2.3 Unidentified Cell types

Reference matrices typically do not cover all cells in a tissue. This could be due to the technical effects or experimenters' decisions depending on the set of cell types that they are interested in. Therefore, we introduce a row in C prior to deconvolution, which leads to estimating an additional column in X' that represents the missing cells. The row C_{UI} that we add to the cellular composition matrix, C, to create

$$C^* = \begin{pmatrix} C \\ C_{UI} \end{pmatrix}$$

is obtained as

$$\sum_{j=1}^{q} c_{j,\cdot} < 1 \qquad \Rightarrow \qquad C_{UI,\cdot} = 1 - \sum_{j=1}^{q} c_{j,\cdot}, \tag{9}$$

see also (Racle et al. 2017; Görtler et al. 2024).

A.2.4 Performance metrics

Let C^{True} be a $q \times n$ matrix containing ground-truth cellular proportions. Mathematically, the cell type-specific performance of a deconvolution tool as introduced in Section 2.2 is defined as

$$R_c(l) := \text{cor}(C_{l,\cdot}, C^{\text{True}}_{l,\cdot}),$$

where l denotes a specific cell type and the correlation is calculated over all samples using only the proportions for that cell type. The sample-specific performance is defined as

$$R_s(m) := \text{cor}\big(C_{\cdot,m}, C^{\text{True}}_{\cdot,m}\big),$$

where m represents a specific sample.

In analogy to (Wang et al. 2019) we combine cell type-specific and sample-specific performance into a single correlation score denoted as R. To do so, we first concatenate the cell type proportions from all cell types into single vectors:

$$\Gamma = \Big(C_{1,\cdot}, C_{2,\cdot}, \cdots, C_{q,\cdot}\Big)^T,$$

$$\Gamma^{\text{True}} = \Big(C^{\text{True}}_{1,\cdot}, C^{\text{True}}_{2,\cdot}, \cdots, C^{\text{True}}_{q,\cdot}\Big)^T,$$

where q denotes the number of cell types. The combined performance is then quantified by the correlation between Γ and Γ^{True}:

$$\text{R} := \text{cor}(\Gamma, \Gamma^{\text{True}}).$$

Additionally, we analyze two absolute error metrics, in analogy to (Wang et al. 2019). The overall root mean squared distance (RMSD) is given by

$$\text{RMSD} := \sqrt{\frac{1}{n \cdot q} \sum_{i=1}^{n \cdot q} (\Gamma_i - \Gamma_i^{\text{True}})^2},$$

and the overall mean absolute deviation (mAD) is defined as

$$\text{mAD} := \frac{1}{n \cdot q} \sum_{i=1}^{n \cdot q} \Big|\Gamma_i - \Gamma_i^{\text{True}}\Big|,$$

where n represents the number of samples.

Complementing the previous cell abundance-centric quality scores we also introduce the bulk-centric quality score

$$\rho(m) := \text{cor}\Big(Y^{\text{rec}}_{\cdot,m}, Y^{\text{obs}}_{\cdot,m}\Big),$$

where m is a sample and Y^{obs} denotes the observed gene expression matrix, measured through experiment, and Y^{rec} is the reconstructed matrix obtained using a reference matrix X and a composition matrix C according to

$$\text{Y}^{\text{rec}}_{\cdot,m} = \sum_{l=1}^{q} X_{\cdot,l}\, C_{l,m}.$$

For Harp, the reconstruction Y^{rec} is performed using X' and C', while for competing tools, the corresponding reference matrices and estimated composition matrices are used.

A.2.5 Digital Tissue Deconvolution (DTD)

To infer the cellular compositions $\hat{C}$ during Harp's *Training* mode (see equation (5)), we use DTD (Görtler et al. 2020). During training of DTD, genes are weighted differently to improve the accuracy of the cell abundance estimation. This method consists of two nested objective functions: an outer function $L(\boldsymbol{g}, \boldsymbol{\alpha})$ and an inner function $\mathcal{L}_{\boldsymbol{g}}$.
For a given vector $\boldsymbol{g}$ of length g, DTD determines $\hat{C}(\boldsymbol{g})$ via

$$\hat{C}(\boldsymbol{g}) = \text{argmin}_{\Gamma} \mathcal{L}_{\boldsymbol{g}}(\Gamma), \tag{10}$$

where

$$\mathcal{L}_{\boldsymbol{g}}(\Gamma) = ||\text{diag}(\boldsymbol{g})(\text{Y} - \text{X}'\Gamma)||_2^2.$$

Here Y is comprised by the bulk samples contained in the training set. The inner function

$$L(\boldsymbol{g}, \boldsymbol{\alpha}) = -\sum_{j=1}^{q} \text{cor}(C'_{j,\cdot}(\boldsymbol{\alpha}), \hat{C}_{j,\cdot}(\boldsymbol{g}))$$

evaluates discrepancies between the cellular frequencies $\hat{C}(\boldsymbol{\alpha})$ estimated in equation (10) and the cellular frequencies $C'(\boldsymbol{\alpha}) = \text{diag}(\boldsymbol{\alpha})\text{C}^*$ given by rescaling the experimental measurements C, with the factors $\boldsymbol{\alpha}$ being given from the previous step of the iterative updating scheme of Harp's *Training* mode, see Section 2.1. Thus, given $\boldsymbol{\alpha}$ we solve

$$\boldsymbol{g}' = \min_{\boldsymbol{g}} L(\boldsymbol{g}, \boldsymbol{\alpha}), \text{ subject to } g_i \geq 0 \text{ and } ||\boldsymbol{g}||_2 = 1. \tag{11}$$

Note that with $\boldsymbol{g}'$, $\beta\boldsymbol{g}'$ is also a minimum of L for any $\beta > 0$. Therefore, the constraint $||\boldsymbol{g}||_2 = 1$ in equation (11) is necessary to ensure uniqueness.

The minimum of $\mathcal{L}_{\boldsymbol{g}}$ is given analytically via

$$\hat{C}(\boldsymbol{g}) = (X'^{\top}\Lambda X')^{-1} X'^{\top}\Lambda Y, \tag{12}$$

where $\Lambda := \text{diag}(\boldsymbol{g})$. Inserting this term into L results in a single optimization problem in $\boldsymbol{g}$, which is minimized using a gradient descent algorithm.
Let $\mu'_j, \hat{\mu}_j$ and $\sigma'_j, \hat{\sigma}_j$ denote the mean and standard deviation of $C'_{j,\cdot}(\boldsymbol{\alpha})$ and $\hat{C}_{j,\cdot}(\boldsymbol{g})$, respectively. The gradient is then computed as

$$\frac{\partial L(\boldsymbol{g}, \boldsymbol{\alpha})}{\partial g_i} = \sum_{j=1}^{q} \sum_{k=1}^{n} \frac{1}{\sigma'_j \hat{\sigma}_j} \Bigg(\frac{\text{cov}(C'_{j,\cdot}(\boldsymbol{\alpha}), \hat{C}_{j,\cdot})}{n\hat{\sigma}_j^2} (\hat{C}_{jk} - \hat{\mu}_j) - \frac{1}{n}(C'_{jk}(\boldsymbol{\alpha}) - \mu'_j) \Bigg) \frac{\partial \hat{C}_{jk}(\boldsymbol{g})}{\partial g_i} \tag{13}$$

with

$$\frac{\partial \hat{C}(\boldsymbol{g})}{\partial g_i} = (X'^{\top}\Lambda X')^{-1} X'^{\top} \delta(i)(1 - X'(X'^{\top}\Lambda X')^{-1} X'^{\top}\Lambda)Y, \tag{14}$$

where $\delta(i) \in \mathbb{R}^{g \times g}$ is defined as

$$\delta(i)_{jk} = \begin{cases} 1 & \text{if } i = j = k \\ 0 & \text{else} \end{cases} . \qquad (15)$$

We emphasize that in the *Training* mode of Harp, DTD is trained on the real data (bulk gene expresssion and cell compositions).

In *Deconvolution* mode, Harp uses the adjusted reference profile matrix X', obtained during training, to deconvolve bulk tissue gene expression data from similar sources (e.g., comparable tissues or profiling technologies) where no experimentally determined tissue composition is available. By default, Harp exploits the adjusted matrix X' in combination with the scaling factor diag($\boldsymbol{g}$) determined in equation (11) in order to solve equation (10), where now Y represents the new bulk samples that need to be deconvolved. For applying DTD in Harp, we used the default setting as advised in the corresponding documentation.

However, the harmonized matrix X' can also serve as a reference profile for use with other deconvolution methods.A.2

A.3 Simulation

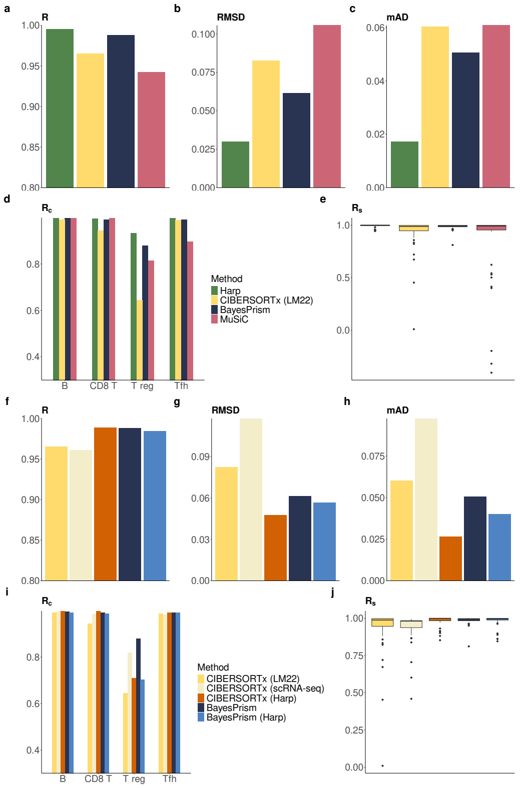

Figure. 8. Evaluation of performance metrics in simulations. (a–e) depict the main benchmark of comparing Harp to the default of competing algorithms, whereas (f–j) depicts the hybrid deconvolution scenario of providing the harmonized reference matrix, X', calculated by Harp to other deconvolution tools. (a–c) and (f–h) show the combined performances R, RMSD, and mAD; (d) and (i) the cell type-specific performance, R_c, and (e) and (j) the sample specific performances. In brackets we notated the exploited reference, where LM22 is the custom reference provided by CIBERSORTx and scRNA-seq refers to the internal CIBERSORTx method of constructing a custom reference from provided scRNA-seq data.

A.3.1 Inter study inconsistencies

As described in Section 3.1 we used the scRNA-seq data from two studies (Steen et al. 2021; Roider et al. 2020) as the sources for our simulations. Notably, we observed various inconsistencies between these studies arising both from technical batch effects and biological variability.

Firstly, these two datasets were processed through different library preparations. The single cell library from Roider et al., (Roider et al. 2020) was prepared by the Chromium single cell v2 3´kit (10x Genomics). Whereas Steen et al., (Steen et al. 2021) employed the 10x Chromium 5´ kit for library preparation. The two assays capture different transcript ends but use the same polydT primer for reverse transcription, see (Hsu et al. 2022). Although they have similar results in cell-type identification, there are some differences in the expression of a certain group of genes (Hsu et al. 2022). The variations in sample preparation protocols between the two studies also cause visible discrepancies between the datasets, see Figure 7a.

Secondly, as expected, we observed patient-specific inter cell-type heterogeneity especially in the malignant B cell compartment, naturally arising from patient-specific mutations in DLBCL cancer, see Figure 7b. We stress that this patient heterogeneity carries essential biological information on the one hand; however, it also makes naive, coarse-grained deconvolution impossible. Thus, preserving this variability in our simulations challenged deconvolution tools to account for patient heterogeneity when predicting cellular composition. In conclusion, by generating artificial bulk mixtures from (Roider et al. 2020) and using (Steen et al. 2021) as single-cell library in simulations, we naturally accounted for various sources of heterogeneity yielding inconsistencies between bulk mixtures and single-cell references that needed to be accounted for during deconvolution.

A.3.2 Preprocessing of single cell data for simulations

The downstream preprocessing of the single cell libraries (Steen et al. 2021) and (Roider et al. 2020) was performed exactly as in (Simeth et al. 2024a)[1], where both datasets were analyzed for their most variable genes, arriving at the joint 1000 most variable genes.

A.3.3 Bulk oversampling

The following oversampling step was included in the generation of pseudo-bulks in order to arrive at a rich collection of artificial bulk mixtures in terms of variation with regard to cellular composition. This approach is analogous to the pseudo-bulk generation in (Chu et al. 2022).

In order to compose a single bulk mixture, we took only a single patient from the given single-cell study in order to keep the patient's cellular *expression* integrity, but we disturbed the cell type-specific *abundance* in order to introduce variance. More precisely, we determined the actual amount of single cells for each cell type within a given patient

$$\begin{pmatrix} \#\text{cells of type A} \\ \#\text{cells of type B} \\ \dots \end{pmatrix},$$

[1] The preprocessed single-cell data is publicly accessible under 10.5281/zenodo.10139153.

**Algorithm**	**R**	**RMSD**	**mAD**	R_s
Harp	**0.998** ± 0.002	**0.017** ± 0.008	**0.011** ± 0.004	**0.997** ± 0.004
BayesPrism	0.975 ± 0.020	0.082 ± 0.027	0.067 ± 0.021	0.955 ± 0.041
MuSiC	0.948 ± 0.033	0.102 ± 0.033	0.067 ± 0.032	0.877 ± 0.057
CIBERSORTx (LM22)	0.970 ± 0.011	0.080 ± 0.015	0.057 ± 0.010	0.936 ± 0.033
CIBERSORTx (scRNA-seq)	0.786 ± 0.151	0.206 ± 0.067	0.165 ± 0.051	0.714 ± 0.141
**Hybrid deconvolution**				
BayesPrism (Harp)	0.985 ± 0.011	0.056 ± 0.019	0.039 ± 0.010	0.969 ± 0.035
CIBERSORTx (Harp)	0.996 ± 0.004	0.027 ± 0.013	0.016 ± 0.007	0.988 ± 0.014
**Uncertain exp. compositions**				
Harp (Distorted)	0.993 ± 0.010	0.030 ± 0.023	0.020 ± 0.013	0.985 ± 0.018
Harp (Cell Type Distorted)	0.998 ± 0.002	0.020 ± 0.007	0.012 ± 0.004	0.993 ± 0.010

**Algorithm/Cell type**	**B**	**CD8 T**	**regulatory T**	**follicular helper T**
Harp	**0.999** ± 0.001	**0.995** ± 0.004	**0.935** ± 0.060	**0.996** ± 0.002
BayesPrism	0.995 ± 0.001	0.914 ± 0.105	0.601 ± 0.309	0.965 ± 0.031
MuSiC	0.999 ± 0.001	0.995 ± 0.003	0.770 ± 0.124	0.901 ± 0.104
CIBERSORTx (LM22)	0.975 ± 0.022	0.934 ± 0.024	0.603 ± 0.333	0.972 ± 0.017
CIBERSORTx (scRNA-seq)	0.976 ± 0.015	0.936 ± 0.072	0.353 ± 0.570	0.872 ± 0.098
**Hybrid deconvolution**				
BayesPrism (Harp)	0.985 ± 0.005	0.938 ± 0.0559	0.621 ± 0.252	0.955 ± 0.040
CIBERSORTx (Harp)	0.999 ± 0.001	0.990 ± 0.015	0.819 ± 0.085	0.994 ± 0.003
**Uncertain exp. compositions**				
Harp (Distorted)	0.993 ± 0.006	0.983 ± 0.020	0.886 ± 0.050	0.993 ± 0.005
Harp (Cell Type Distorted)	0.999 ± 0.001	0.994 ± 0.008	0.928 ± 0.064	0.996 ± 0.002

Table 1. Performance metrics evaluated across five simulation runs for all deconvolution benchmarks. Harp was trained on 20 samples and, along with other methods, tested on 40 samples. Performance improvements for each algorithm when using Harp's reference are shown in blue. The highest performance in the main deconvolution benchmark is highlighted in bold.

**Algorithm**	BayesPrism	MuSiC	CIBERSORTx (Harp)
Harp	4.6×10^{-4}	3.1×10^{-5}	4.6×10^{-4}
**Hybrid deconvolution**			
CIBERSORTx (Harp)	8.0×10^{-2}	2.0×10^{-2}	–
**Uncertain exp. compositions**			
Harp (Cell Type Distorted)	1.3×10^{-5}	4.8×10^{-7}	–
Harp (Distorted)	9.3×10^{-1}	7.9×10^{-1}	–

**Algorithm**	BayesPrism	MuSiC	CIBERSORTx (Harp)
Harp	1.8×10^{-9}	4.9×10^{-13}	1.8×10^{-9}
**Hybrid deconvolution**			
CIBERSORTx (Harp)	1.6×10^{-5}	3.4×10^{-8}	–
**Uncertain exp. compositions**			
Harp (Distorted)	6.0×10^{-3}	8.4×10^{-5}	–
Harp (Cell Type Distorted)	2.2×10^{-11}	2.6×10^{-15}	–

Table 2. Z-test statistics on Fisher's transformed cell-type wise (top table) and sample wise (bottom table) correlation coefficients of Harp against the second best performing methods on simulated data. The algorithm indicated by the the table's row is tested for significance against the algorithm indicated by the column. Results are always shown for the first run.

and then perturbed with a normally distributed multiplicative factor given via

$$|\min(\mathcal{N}(1,1), 1.5)|, \tag{16}$$

i.e., we arrive at the perturbation

$$\begin{pmatrix} |\min(\mathcal{N}(1,1), 1.5)| \cdot \#\text{cells of type A} \\ |\min(\mathcal{N}(1,1), 1.5)| \cdot \#\text{cells of type B} \\ \dots \end{pmatrix}.$$

Here we limited to a maximal factor of 1.5 in order to keep oversampling on a moderate scale, but still introduce suitable

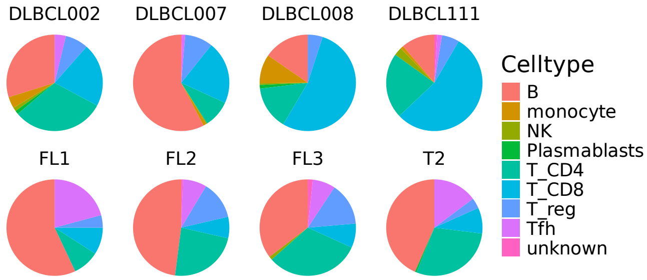

(a) Patients in Steen et al.

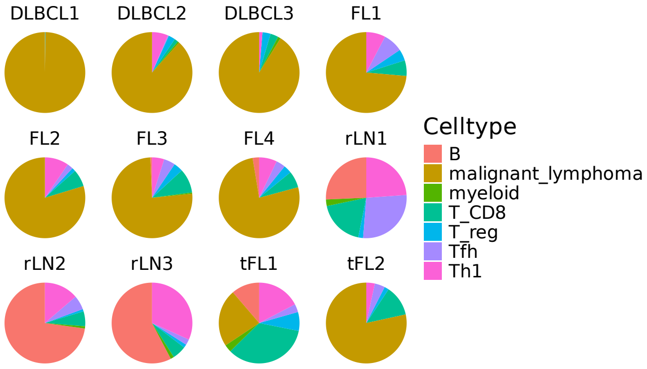

(b) Patients in Roider et al.

Figure. 9. Cellular composition of the patients from Steen et al. and Roider et al. The lymphoma entities comprise Diffuse Large B-cell Lymphoma (DLBCL), Follicular Lymphoma (FL), Transformed Follicular Lymphoma (tFL), Tonsilitis (T) and Reactive lymph nodes (rLN)

variance. This approach results in the total amount of cells to be randomly sampled for each cell type out of the given patient. Thus, we arrived at an artificial bulk mixture $Y_{\cdot,s}$ by summing over all sampled single-cell profiles. Note that here we sampled with replacement, in order to be able to satisfy multiplicative factors larger than one in equation (16). Note that this approach naturally provides us with ground truth cellular proportions, as also explained in (Wang et al. 2019, Methods) and (Chu et al. 2022, Supplementary Note 4). More precisely, the ground truth cell type abundance in bulk sample m of cell type l can be computed as

$$\frac{\sum_{i=1}^{g} Y_{i,m}(l)}{\sum_{i=1}^{g} Y_{i,m}},$$

where $Y_{\cdot,m}(l)$ denotes the cell type-specific expression of cell type l in artificial bulk sample m and $Y_{\cdot,m}$ denotes the total expression of artificial bulk sample m.

Representative pseudo-bulk samples and the distribution of cellular abundance across all pseudo bulks are depicted in Figures 11.

Note that we performed five independent simulation runs, thus, the random cell sampling for bulk composition is different in each run.

A.3.4 Gene distortion

In order to exaggerate local inconsistencies, see Section 1, we randomly distorted a fixed amount of genes within the simulated bulk mixtures by a multiplicative factor. This approach is analogous to the linear multiplicative noise model in (Chu et al. 2022). Therefore, we fixed the percentage of genes we want to distort in order to arrive at a set $\mathcal{G}$ of genes to be distorted. In our case we configured this to be 40% of the 1000 genes available. For each gene $g \in \mathcal{G}$ we determined a multiplicative distortion factor ζ_g by drawing from the normal distribution,

$$\zeta_g \in |\mathcal{N}(1, 5)|.$$

The distribution parameters are chosen to yield biologically reasonable fold changes. This approach provides us with distorted bulk samples by multiplying

$$\zeta_g Y_{g,\cdot}$$

for all $g \in \mathcal{G}$. In particular this means that each gene is distorted constantly across bulk samples in order to simulate technical gene-specific batch effects in gene sequencing measurements.

Note that we performed five independent simulation runs, thus, the distortion factors are different in each run.

A.3.5 Harp generalizes stably for sufficient amount of training samples

As an initial benchmark we explored Harp's performance in terms of variable training cohort sizes. Therefore, we generated a total set of 160 bulk mixtures available for training and 40 test bulks. Out of the set of training bulks we subsampled a variable amount of bulk mixtures in the range [6, 160], trained Harp and evaluated its performance on the 40 held-out test samples. The results of this benchmark are depicted in Figure 10. As expected, all quality scores showed consistently inferior results for insufficient training samples and stabilize at competitive values after enough samples were added to the training set. We observed that this stabilization occurred at around 50 training samples, leading to the selection of this cohort size for remaining simulation benchmarks.

A.3.6 Configurations of competing methods for benchmarking deconvolution tools on simulated data

CIBERSORT (Newman et al. 2015) is designed to use a given reference in deconvolution, and comes with the custom reference LM22 derived from microarray data, see (Newman et al. 2015). Its successor, CIBERSORTx (Newman et al. 2019), provides the option to learn a custom reference from given single-cell or sorted RNA-seq data. Furthermore, CIBERSORTx addresses cross-platform variation via refined batch correction. Therefore, the authors introduced bulk mode (B-mode) and single-cell mode (S-mode) batch correction. In our simulations, as the employed single-cell dataset (Steen et al. 2021) was generated using 10x Chromium, see Section A.3.1, we followed the authors' suggestion and applied S-mode batch correction when providing CIBERSORTx with the single-cell dataset. We denote this version of CIBERSORTx as CIBERSORTx (scRNA-seq). For the microarray reference LM22 we followed the authors' suggestion and applied B-mode batch correction. We denote this version of CIBERSORTx as CIBERSORTx (LM22) in our evaluations. Additionally, instead of the generic microarray reference LM22 we also provided the

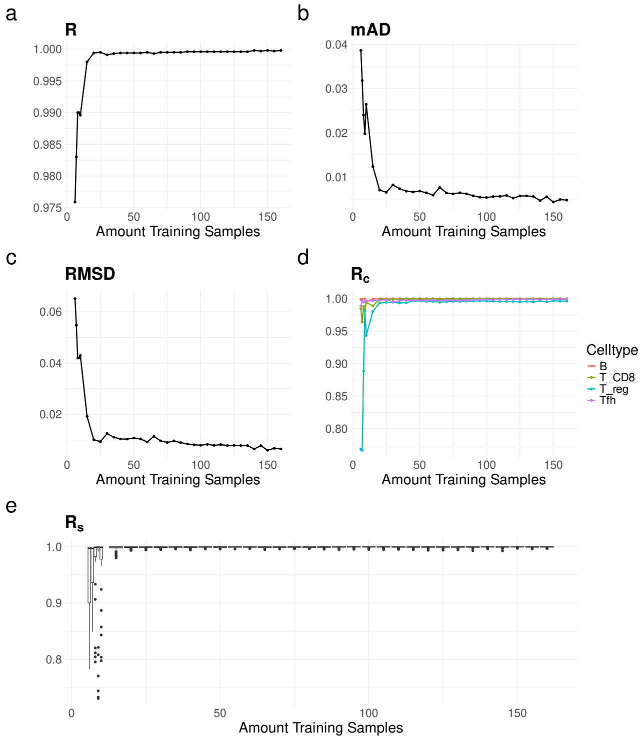

Figure. 10. Impact of the amount of artificial bulk samples used for training Harp on main performance metrics.

reference learned by Harp to CIBERSORTx. We note that due to Harp's capability of adapting its reference to the training bulks we did not apply any of the proposed batch corrections within the CIBERSORTx algorithm on the simulated data in order to underline the fact that our reference already overcame these batch effects by integrating bulk information during training. We denote this approach as CIBERSORTx (Harp). Note, that when employing LM22 we mapped the granular cell types of this reference to the cell compartments analyzed in simulations as follows: naive and memory B cells were mapped to B cells. Naive, memory resting and memory activated CD4 T cells were mapped to CD 4 T cells.

Concerning BayesPrism, the standard algorithm exploits fine-grained cellular states in order to optimally integrate single-cell information, see (Chu et al. 2022). Therefore, we performed automated subclustering interfaced in the Seurat package (Stuart et al. 2019) on the given single-cell library. This provided us with cellular states that refined the original cell types. The subclustering information and the single-cell library were then provided to BayesPrism. This standard usage of BayesPrism is denoted as BayesPrism in our benchmarks. Similarly to CIBERSORTx we also evaluated the performance of BayesPrism when provided with the Harp reference, which is possible as BayesPrism allows for replacing subclustering and single-cell information by directly providing a reference by choosing the input.type = "GEP", see also the corresponding vignette.[2] This approach is referred to as BayesPrism (Harp) in the following. However, we note that the recommended default usage of BayesPrism is to receive a rich scRNA-seq count matrix in order to internally derive a reference via maximum likelihood estimation, see (Chu et al. 2022). For applying MuSiC (Wang et al. 2019) we used the default setting as advised in the corresponding documentations. MuSiC does not allow for providing a custom reference as an entire single cell library is required for inference. Note that there exists also a more recent version MuSiC2 (Fan et al. 2022) which is equivalent to the initial MuSiC implementation in our usecase, as we do not include different sample conditions.

A.3.7 Statistical testing

To evaluate whether correlation coefficients computed by Harp posed significant improvements over competing algorithms, we followed the approach described in Section "Statistical test on cell type level Pearson correlation coefficients" of (Chu et al. 2022). There a one sided z-test is performed on cell type level correlation coefficients, that tests wether these coefficients as calculated by BayesPrism are significantly greater (in mean across cell types) than another competing deconvolution tool. To do so, z values are computed from correlation coefficients by applying Fisher's Z-transformation. We always compared Harp and its derivations with the second best performing method. As on simulated data for some metrics BayesPrism and for some MuSiC was the second best performing method, we compared to those methods. Besides the assessment of cell-type specific correlation coefficients R_c we also performed this z-test on sample specific correlations R_s.

A.3.8 Statistical Analysis

We ran both simulation and real world evaluation benchmarks over five independent runs in order to arrive at independent models on varying data in order to compute mean performance and report standard deviation across runs.

For simulations this means that random splitting of patients in train and test cohort, the sampling of cells, the gene distortion factors and the proportions distortion factors change in each run. For simulations the results are shown in Table 1. We see that Harp outperformed its competitors in all evaluation metrics. When results are plotted we only depict the first run, see Figures 8 and 12.

The statistical test, which was discussed in Section A.3.7, was applied to the simulated data. The results are reported in Table 2 and for the first run of our experiments. On simulated data using ground truth proportions in Harp we see from Table 2 that correlation coefficients pose significant improvements over competing methods.

For real data, for the deconvolution of bulk RNA-seq expression data, we randomly selected 150 samples for training Harp, and 100 samples for testing Harp as well as all other algorithms. When using the sorted RNA-seq reference, Table 3 shows that, in terms of overall performance—measured by R, RMSD, and mAD—as well as sample-specific correlation (R_s), Harp outperformed both BayesPrism and CIBERSORTx. However, for cell type-specific correlation, CIBERSORTx (LM22) achieved the best performance among all algorithms. In predicting bulk gene expression profiles, Harp outperformed CIBERSORTx, while BayesPrism achieved the best performance with a correlation of 1. In hybrid deconvolution, the learned reference from Harp improved the overall and sample-specific performance of both methods. However, it had a negative impact on cell type-specific

[2] The BayesPrism vignette is provided under https://github.com/Danko-Lab/BayesPrism.

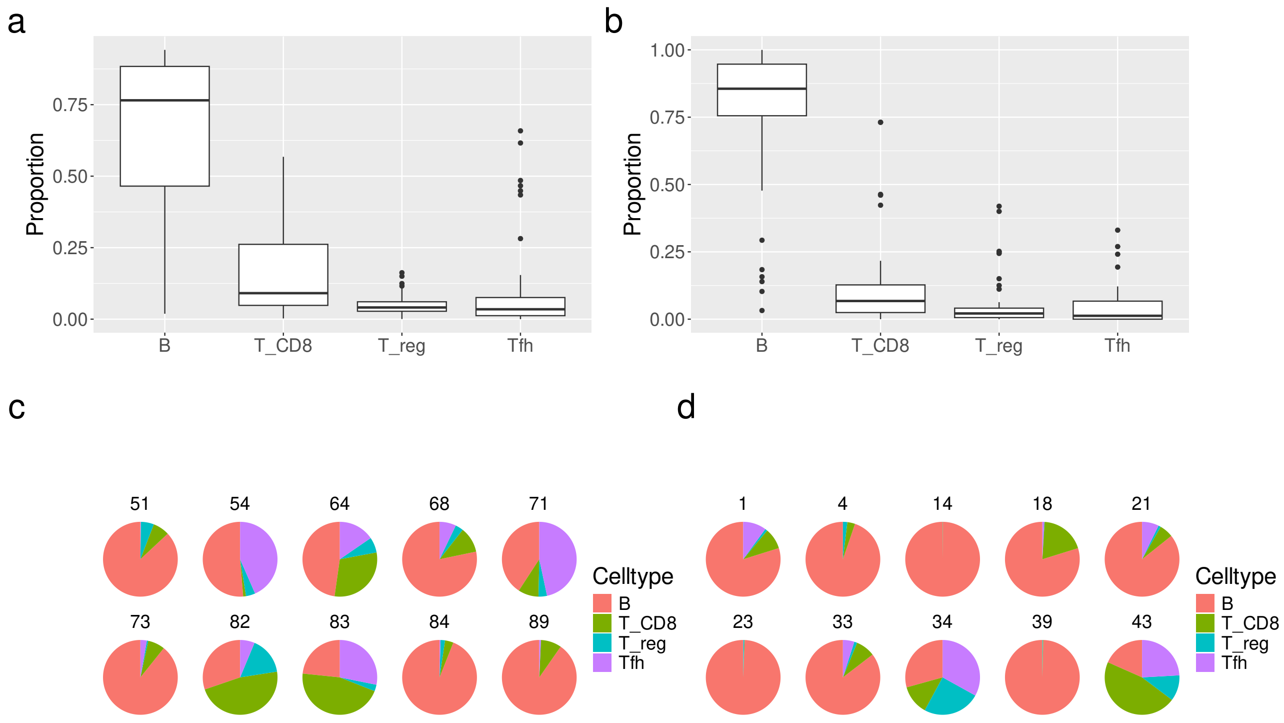

Figure. 11. Overview of cellular composition for a selection of test bulk samples (a, c) and training bulk samples (b, d) used in simulations.

performance, except for Monocytes and Plasmablasts. This reference also had a positive impact on the prediction of bulk gene expression profiles when used with CIBERSORTx.

We followed the same procedure for deconvolution of bulk RNA-seq expression data using a microarray-based reference. Table 6 shows that Harp achieved better results than the other algorithms across all performance metrics, except in the prediction of B cell populations and bulk gene expression profiles, where BayesPrism achieved the best results. In hybrid deconvolution, the reference learned by Harp improved overall performance, sample-specific performance, bulk gene expression prediction (where improvement was possible, as BayesPrism already had a correlation of 1), and the prediction of Monocyte and T cell populations for both methods.

For visualization, we selected runs that were most representative of the average performance (see Figure 5, 6, 17 and 18). The Z-test scores for real data, presented in Table 4 and Table 7, were derived from the results of these selected runs. Due to the variability in cell type-specific performance across algorithms, we compared Harp with both BayesPrism and CIBERSORTx.

When deconvolving bulk RNA-seq data using a sorted RNA-seq reference, Harp significantly improved sample-specific performance compared to both methods, but did not yield significant gains in cell type-specific accuracy. In hybrid deconvolution, we found that the reference learned by Harp significantly improved the sample-specific performance of both methods, while no improvements were observed in cell type-specific performance (Table 3).

Table 6 shows that deconvolution of bulk RNA-seq data using a microarray-based reference produced a similar pattern of results to those obtained with a reference derived from sorted RNA-seq data. Additionally, in this case, using the Harp reference did not lead to a significant improvement in either cell type-specific or sample-specific correlation for CIBERSORTx, compared to its customized reference based on sorted RNA-seq data.

As another real-world example, we performed a deconvolution benchmark on microarray-based bulk gene expression data using a microarray-derived reference (for more details on the data, see Section A.5.4). We randomly selected 12 samples for training Harp and eight samples for the test set. This selection process was repeated five times. The analysis and results are reported in Section A.5. We also conducted a statistical test on one representative run, selected based on overall performance. The results show that Harp achieved a significant improvement in sample-specific performance over CIBERSORTx (LM22), while no significant improvement was observed in cell type-specific correlation compared to competing methods (see Table 12). However, in this scenario, all methods exhibited high standard deviation in cell type-specific correlation, making it unclear if the non-significant differences are due to actual performance similarity.

According to Figure 10, simulations indicated that 20 training samples were sufficient to train Harp. In this simulation setting, the test sample size was 40, and the data included four distinct cell types. To assess the performance of Harp with only 20 training samples on real data, we conducted an additional analysis. Using bulk RNA-seq data and two different reference datasets (sorted RNA-seq and microarray), we ran the benchmark five times for each reference. In each run, 20 samples were randomly selected to train Harp, and 100 samples were used to test Harp as well as all other algorithms.

All performance metrics were evaluated, and the results are presented in Table 5 and Table 8 (see Section A.4.2 for further details).

Using the microarray-based reference, Table 8 shows that Harp's performance across nearly all metrics remained comparable to results obtained with 150 training samples. However, in this setting, Harp did not achieve the best performance in predicting T cell and NK cell populations. Specifically, BayesPrism performed best for T cell prediction, while CIBERSORTx (LM22) outperformed other methods in NK cell prediction. In hybrid deconvolution, the reference learned by Harp continued to enhance both methods in terms of overall, sample-specific, and bulk gene expression performance (where improvement was possible—BayesPrism had already achieved a correlation of 1). However, no gains were observed in cell type-specific performance, except for BayesPrism in the prediction of Monocytes.

When using a reference from RNA-seq technology, Table 5 shows that Harp's overall performance, sample-specific performance, and bulk gene expression prediction were also comparable to those obtained using 150 training samples (Table 3). Among all evaluated metrics, Harp achieved the best performance, except for overall correlation (R) and bulk gene expression prediction (ρ), where BayesPrism performed the best. However, reducing the number of training samples to 20 had a negative impact on cell type specific performance of Harp.

A.3.9 Hybrid Deconvolution

One might argue that, in the main simulation benchmark of Section 3.1.2, Harp had an advantage, because it was trained with additional data, including ground-truth compositions, and could perform data harmonization—a capability not available to the competing algorithms. To test whether harmonization could also improve the performance of these methods, we provided the harmonized reference matrix, X', calculated by Harp to competing deconvolution tools. We then evaluated these hybrid methods, which combine Harp's harmonized reference X' with the deconvolution algorithms of CIBERSORTx and BayesPrism. Figure 8 shows that the harmonized reference learned by Harp is beneficial for deconvolution with CIBERSORTx. In particular, using Harp's reference significantly improved all performance scores compared to both the default LM22 and customized scRNA-seq references. Interestingly, hybrid CIBERSORTx with the Harp reference outperformed BayesPrism, even though BayesPrism leverages full single-cell information. For BayesPrism with the Harp reference, we did not observe a general improvement; however, performance scores, including sample-specific correlations, indicate that its performance is on par with that of CIBERSORTx (LM22). As observed previously, a notable strength of the Harp reference is its performance in the lowly abundant regulatory T cell compartment. In this case, using a generic reference (CIBERSORTx (LM22)) or relying solely on single-cell information (BayesPrism and CIBERSORTx (scRNA-seq)) appears to miss the fine-grained cellular signals present in the bulk expression data.

Table 1 shows CIBERSORTx (Harp) and BayesPrism (Harp) outperforms the respective classical methods in all quality scores, expect for B and follicular helper T cell-specific correlation in the case of BayesPrism (Harp). Table 2 shows that correlation coefficients of CIBERSORTx (Harp) in the case of outperformance are significantly better than the second best methods BayesPrism and MuSiC.

A.3.10 Simulating uncertain experimental compositions

As discussed in Section 2.1 flow cytometry measurements suffer from cell type specific bias. Mathematically, we simulated this scenario, by drawing a *cell type-specific distortion rate*, $\boldsymbol{\delta}(l)$ (with l representing a cell type), from the distribution

$$\max\{0.2, \min\{1.0, |\mathcal{N}(0.5, 0.2)|\}\}.$$

This means, that we limited $\boldsymbol{\delta}(l)$ in [0.2,1] in order to arrive at biologically reasonable distortions, and allow for a mean loss of 50% within a cell type with a standard deviation of 0.2. These factors were used to simulate compromised proportion measurements via

$$C := \text{diag}(\boldsymbol{\delta})\text{C}_{\text{True}},$$

where C_{True} is a $q \times n$ matrix holding the ground-truth cell type proportions for each sample.

We denote the model trained on these compromised proportions as Harp (Cell Type Distorted). As expected, Figure 13 reveals, that the cell specific correction rates $\boldsymbol{\alpha}$ indeed captured the simulated distortion by satisfying the relation $\boldsymbol{\alpha}(l) \approx \boldsymbol{\delta}^{-1}(l)$.

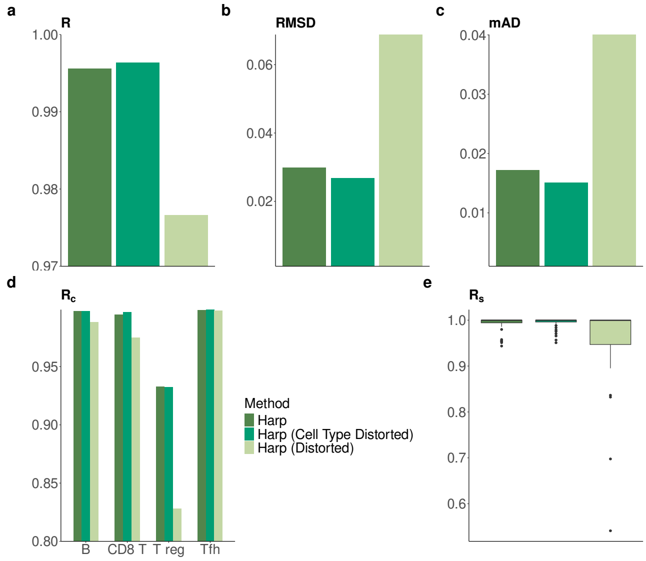

Figure. 12. Comparison of Harp when provided with correct cellular proportions vs. distorted proportions. The distorted case is indicated by (Distorted). (a–c) show the combined performances R, RMSD and mAD; (d) represents the cell type-specific performance , R_c; and (e) depicts the sample specific performances, R_s.

A cell type-specific distortion rate however might be oversimplifying the actual bias present in cell composition measurements. Thus, we also multiplied ground truth proportions by a factor drawn from the distribution

$$\max\{0.2, \min\{1.0, |\mathcal{N}(0.9, 0.2)|\}\}.$$

Note that as opposed to the previous approach, this factor now varies across cell-types as well as samples. We denote the model trained on these compromised proportions as Harp (Distorted)

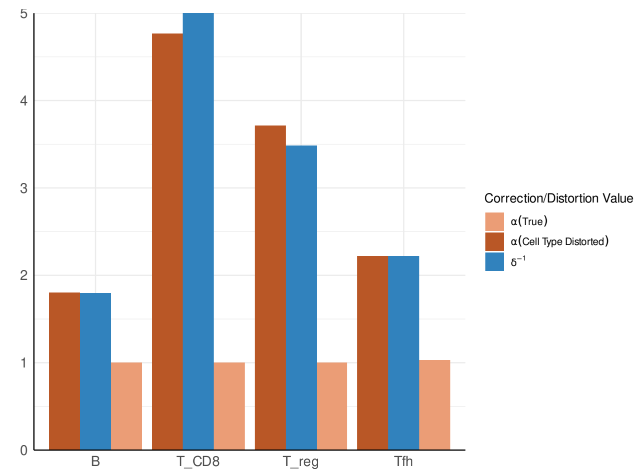

Figure. 13. Cell type-specific correction values, $\boldsymbol{\alpha}$, computed by Harp when provided with correct cell proportions (True) and distorted proportions (Distorted) in *Training* mode, respectively. These values are compared to the inverse distortion rate $\boldsymbol{\delta}^{-1}$.

Table 1 shows that Harp (Cell Type Distorted) outperforms the respective classical methods in all quality scores and Table 2 shows that correlation coefficients are significantly better than the second best methods BayesPrism and MuSiC. For the more extreme case of Harp (Distorted) we notice that in most of the quality metrics there is an improvement compared to other deconvolution tools, however the cell type specific correlation gains are overall not significant. Nevertheless, the sample wise correlation gains are still significant.

A.3.11 Harp's regularization approach balances deconvolution accuracy and biological integrity

In order to understand Harp's dependency on the hyperparameter λ, see Sections A.2.2 and 2.1, we analyzed its evolution during cross-validation in the *Training* mode of our algorithm and we studied the quality of models fitted for different values of λ. Therefore, we used the exact same simulated data as in the main benchmark in Section 3.1.2.

Concerning the first point, the correlation computed for each candidate λ in the cross-validation phase is shown in Figure 14. As expected, we observed optimal performance for an intermediate $\lambda' = 0.8$. This is due, on the one hand, to the fact that the naive unregularized solution ($\lambda = 0$) is expected to overfit to the training set and, therefore, cannot optimally explain the held-out samples. On the other hand, excessively high values of λ prevent the incorporation of bulk information by sticking to the anchor X^*. This explains why we observed a steep decline after the optimal λ' was reached. However, we stress that during cross-validation Harp exploits only a simple least square regression to arrive at a rough estimate of cellular abundance, see Section A.2.2.

In Figure 14 (a) we show the trajectory in the first iteration of Harp's *Training* mode, i.e., where X' in equation (4) is determined for $\boldsymbol{\alpha}$ being the identity matrix. In Figure 14 (b), however, we show the trajectory in the second iteration, i.e., subsequent to determining $\boldsymbol{\alpha}$ in equation (5). As expected both figures appear identical, because the simulated data uses ground truth proportions for C and thus, the $\boldsymbol{\alpha}$ determined in equation (5) is still an approximation of the identity matrix, see also $\boldsymbol{\alpha}$(True) in Figure 13.

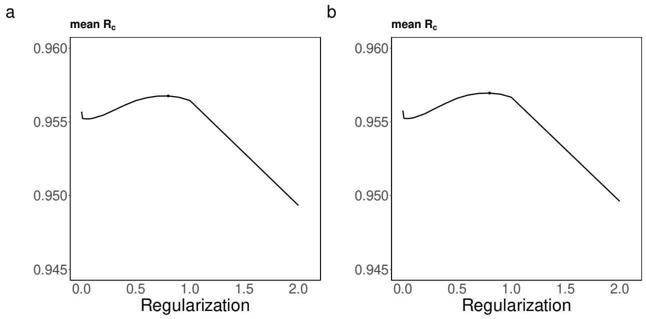

Figure. 14. Evolution of λ during cross-validation in Harp's *Training* mode. The optimum is marked as a point. (a) shows the trajectory before cell type-specific correction with α and (b) in the second iteration of our proposed updating scheme, i.e., after this correction step.

Next, we had a closer look at the performance of the *entire* Harp algorithm for different λ values, see Figure 15. A major difference to the previous paragraph is thus, that DTD is used during *Deconvolution* mode, which is far more elaborate than plain least squares regression. Here, we represent the R_c metric and the R_s metric for varying values of λ, see Section 2.2. Regarding cell type-specific performance (R_c), we observed that the cell types were generally well captured for suitably small values of λ and the optimal lambda value is in the interval $[0.5, 0.75]$ which is a regime consistent with the optimal cross-validated $\lambda' = 0.8$ from the previous paragraph. However, for values close to zero, R_c behaved instable for regulatory T cells indicating that this regularization regime gives suboptimal deconvolution results. This again underlines that lowly abundant cell compartments are especially challenging in deconvolution, as already noted in Section 3.1.2. Interestingly, R_s metric was not prone to instabilities for λ values close to zero. This also indicates that tuning λ during cross-validation only to optimize the R_c metric, see Section A.2.2, is natural, because the R_s metric is stable independently of λ, at least in our simulation scenario. Considering a broader spectrum of λ values, Figures 15 (b) and 15 (d) show that both metrics broke down in the regime of very high regularization, indicating that sticking with the naive single-cell reference hinders the adequate integration of bulk information in the *Training* mode of Harp.

A.4 Data harmonization with Harp improved deconvolution accuracy in a study combining data from two distinct sources

A.4.1 Data processing

For this case study the bulk gene expression and flow cytometry data were given by (Zimmermann et al. 2016). We utilized TPM-normalized gene expression data and cell abundances obtained from BostonGene[3], provided as part of the Kassandra project (Zaitsev et al. 2022). The cell types included in the flow cytometry data were labeled as B Naive, B Ex, B NSM,

[3] The RNA-seq expression and flow cytometry data are also available under the ID sdy67 on BostonGene (https://science.bostongene.com).

Algorithm/ Metric	R	RMSD	mAD	R_s	ρ
Harp	**0.955** $\pm$ 0.012	**0.078** $\pm$ 0.016	**0.058** $\pm$ 0.013	**0.966** $\pm$ 0.013	0.976 $\pm$ 0.002
BayesPrism	0.931 $\pm$ 0.004	0.099 $\pm$ 0.002	0.077 $\pm$ 0.001	0.940 $\pm$ 0.005	**1.00** $\pm 1.00 \times 10^{-6}$
CIBERSORTx (LM22)	0.734 $\pm$ 0.022	0.163 $\pm$ 0.006	0.121 $\pm$ 0.004	0.729 $\pm$ 0.018	0.894 $\pm$ 0.003
**Hybrid deconvolution**					
BayesPrism (Harp)	0.950 $\pm$ 0.006	0.0877 $\pm$ 0.006	0.069 $\pm$ 0.006	0.961 $\pm$ 0.005	$1.00 \pm 1.06 \times 10^{-6}$
CIBERSORTx (sorted RNA-seq)	0.911 $\pm$ 0.004	0.113 $\pm$ 0.003	0.094 $\pm$ 0.003	0.919 $\pm$ 0.006	0.954 $\pm$ 0.002
CIBERSORTx (Harp)	0.918 $\pm$ 0.020	0.098 $\pm$ 0.010	0.076 $\pm$ 0.007	0.936 $\pm$ 0.016	0.987 $\pm$ 0.003

Algorithm/ Cell type	Monocytes	T cells	NK cells	B cells	Plasmablasts
Harp	**0.730** $\pm$ 0.045	0.665 $\pm$ 0.046	0.534 $\pm$ 0.283	0.433 $\pm$ 0.201	0.465 $\pm$ 0.138
BayesPrism	0.716 $\pm$ 0.063	0.644 $\pm$ 0.0301	0.699 $\pm$ 0.042	0.244 $\pm$ 0.045	0.442 $\pm$ 0.269
CIBERSORTx (LM22)	0.698 $\pm$ 0.044	**0.678** $\pm$ 0.028	**0.704** $\pm$ 0.056	**0.670** $\pm$ 0.035	–
**Hybrid deconvolution**					
BayesPrism (Harp)	0.718 $\pm$ 0.048	0.618 $\pm$ 0.054	0.500 $\pm$ 0.100	-0.001 ± 0.027	0.547 $\pm$ 0.140
CIBERSORTx (sorted RNA-seq)	0.492 $\pm$ 0.082	0.444 $\pm$ 0.075	0.531 $\pm$ 0.061	0.260 $\pm$ 0.044	0.268 $\pm$ 0.177
CIBERSORTx (Harp)	0.717 $\pm$ 0.048	0.554 $\pm$ 0.094	0.238 $\pm$ 0.097	0.246 $\pm$ 0.151	0.395 $\pm$ 0.189

Table 3. Evaluation of performance metrics for RNA-seq deconvolution using a sorted RNA-seq reference across five runs on 100 validation samples, for both the main and hybrid deconvolution benchmarks. Harp was trained on 150 training samples. Performance improvements for each algorithm when using Harp's reference are shown in blue. The best performance in the main benchmark is highlighted in **bold**.

Algorithm	BayesPrism	CIBERSORTx (LM22)	CIBERSORTx (sorted RNA-seq)	BayesPrism (Harp)	CIBERSORTx (Harp)
Harp	0.525	0.994	9.9×10^{-6}	0.023	0.052
**Hybrid deconvolution**					
BayesPrism (Harp)	0.812	1.000	0.102	–	0.083
CIBERSORTx (Harp)	0.989	1.000	0.547	0.917	–

Algorithm	BayesPrism	CIBERSORTx (LM22)	CIBERSORTx (sorted RNA-seq)	BayesPrism (Harp)	CIBERSORTx (Harp)
Harp	0.003	7.3×10^{-17}	2.5×10^{-7}	0.224	0.001
**Hybrid deconvolution**					
BayesPrism (Harp)	0.002	4.3×10^{-20}	3.2×10^{-7}	–	3.6×10^{-5}
CIBERSORTx (Harp)	0.847	1.5×10^{-10}	0.163	1.000	–

Table 4. Z-test statistics on Fisher's transformed cell type-wise, R_c, (top table) and sample-wise, R_s, (bottom table) correlation coefficients of Harp against other methods on RNA-seq data using a RNA-seq reference data. The algorithm indicated by the table's row is tested for significance against the algorithm indicated by the column. Results are shown for the most representative run (i.e., one similar to the mean performance).

B SM, Plasmablasts, CD4 T cells, CD8 T cells, Basophils LD, Dendritic cells, Plasmacytoid dendritic cells, Monocytes C, Monocytes I, Monocytes NC, NK cells, B Memory, T cells, Monocytes C+I, Monocytes NC+I, Monocytes, B cells, and Lymphocytes. Since not all cell types were present in every sample, we included only those samples that contained all specified cell types and were paired with bulk RNA-seq data. This filtering resulted in a final dataset comprising 250 samples, which was then randomly divided into a 150-sample training set and a 100-sample test set. We repeated the random split five times and conducted statistical analyses. Figure 5, 6, 17, 18, Table 9 and 10 were generated using the results from the most representative run. To further validate Harp, we repeated the same procedure using only 20 training samples and 100 test samples (see Section A.3.8). To evaluate the tainting of Harp in these scenarios, we performed 3-fold cross-validation for the RNA-seq reference and 4-fold cross-validation for the microarray reference, due to the limited number of training samples.

The cell types included in the data represented different levels of cell type granularity. For further analysis, we selected B cells (B memory cells and B naive cells), Monocytes, T cells, NK cells, Plasmablasts, Basophils LD, Plasmacytoid dendritic cells, and Dendritic cells. The sum of the cell type populations selected for each sample did not add up to 100%, The cell proportions across samples averaged 95.99%, with values ranging from a minimum of 85.98% to a maximum of 99.98%. Therefore, we added an additional row to the cellular composition matrix to account for the Unidentified cells, see Section A.2.3. To align the flow cytometry data with our references (both sorted RNA-seq and microarray) and/or because of the rarity of certain cell types, we also categorized Basophils LD, Plasmacytoid dendritic cells, and Dendritic cells under the "Unidentified" category in our analysis.

Algorithm/ Metric	R	RMSD	mAD	R_s	ρ
Harp	0.923 ± 0.013	**0.0934** ± 0.007	**0.072** ± 0.005	**0.942** ± 0.011	0.973 ± 0.006
BayesPrism	**0.929** ± 0.008	0.100 ± 0.004	0.077 ± 0.003	0.939 ± 0.008	**1.00** ± 1.35 × 10^{-6}
CIBERSORTx (LM22)	0.729 ± 0.010	0.165 ± 0.002	0.121 ± 0.003	0.728 ± 0.007	0.895 ± 0.002
**Hybrid deconvolution**					
BayesPrism (Harp)	0.960 ± 0.005	0.072 ± 0.008	0.055 ± 0.007	0.970 ± 0.005	1.00 ± 1.4 × 10^{-6}
CIBERSORTx (sorted RNA-seq)	0.918 ± 0.007	0.109 ± 0.005	0.0905 ± 0.004	0.926 ± 0.006	0.953 ± 0.002
CIBERSORTx (Harp)	0.949 ± 0.011	0.082 ± 0.006	0.063 ± 0.004	0.965 ± 0.007	0.987 ± 0.003

Algorithm/ Cell type	Monocytes	T cells	NK cells	B cells	Plasmablasts
Harp	0.653 ± 0.049	0.474 ± 0.165	0.175 ± 0.043	0.008 ± 0.094	0.326 ± 0.224
BayesPrism	0.675 ± 0.040	0.652 ± 0.059	0.672 ± 0.046	0.344 ± 0.229	0.487 ± 0.244
CIBERSORTx (LM22)	**0.693** ± 0.044	**0.685** ± 0.085	**0.715** ± 0.0143	**0.669** ± 0.091	–
**Hybrid deconvolution**					
BayesPrism (Harp)	0.703 ± 0.075	0.600 ± 0.050	0.404 ± 0.056	0.192 ± 0.134	0.424 ± 0.293
CIBERSORTx (Harp)	0.630 ± 0.122	0.479 ± 0.186	0.308 ± 0.027	0.145 ± 0.190	0.373 ± 0.174
CIBERSORTx (sorted RNA-seq)	0.516 ± 0.065	0.484 ± 0.059	0.505 ± 0.065	0.289 ± 0.120	0.280 ± 0.176

Table 5. Evaluation of performance metrics for RNA-seq deconvolution using a sorted RNA-seq reference across five runs on 100 validation samples, for both the main and hybrid deconvolution benchmarks. Harp was trained on 20 training samples. Performance improvements for each algorithm when using Harp's reference are shown in blue. The best performance is highlighted in **bold**.

Algorithm/ Metric	R	RMSD	mAD	R_s	ρ
Harp	**0.943** ± 0.012	**0.080** ± 0.008	**0.057** ± 0.007	**0.941** ± 0.014	0.925 ± 0.009
BayesPrism	0.764 ± 0.024	0.154 ± 0.007	0.117 ± 0.006	0.759 ± 0.030	**1.00** ± 6.1 × 10^{-7}
CIBERSORTx (LM22)	0.734 ± 0.022	0.163 ± 0.006	0.121 ± 0.004	0.729 ± 0.018	0.894 ± 0.003
**Hybrid deconvolution**					
BayesPrism (Harp)	0.929 ± 0.010	0.089 ± 0.006	0.066 ± 0.004	0.931 ± 0.014	1.00 ± 6.1 × 10^{-7}
CIBERSORTx (Harp)	0.935 ± 0.012	0.088 ± 0.006	0.066 ± 0.005	0.938 ± 0.014	0.937 ± 0.013

Algorithm/ Cell type	Monocytes	T cells	NK cells	B cells
Harp	**0.746** ± 0.051	**0.757** ± 0.056	**0.749** ± 0.093	0.681 ± 0.048
BayesPrism	0.705 ± 0.050	0.694 ± 0.019	0.676 ± 0.047	**0.691** ± 0.052
CIBERSORTx (LM22)	0.698 ± 0.044	0.678 ± 0.028	0.704 ± 0.060	0.670 ± 0.035
**Hybrid deconvolution**				
BayesPrism (Harp)	0.729 ± 0.056	0.703 ± 0.038	0.619 ± 0.055	0.673 ± 0.060
CIBERSORTx (Harp)	0.738 ± 0.043	0.683 ± 0.067	0.513 ± 0.102	0.618 ± 0.093

Table 6. Evaluation of performance metrics for RNA-seq deconvolution using a microarray-based reference across five runs on 100 validation samples, for both the main and hybrid deconvolution benchmarks. Harp was trained on 150 training samples. Performance improvements for each algorithm when using Harp's reference are shown in blue. The best performance is also highlighted in **bold**.

The sorted RNA-seq signature PBMC data was obtained from four healthy donors, as reported in (Monaco et al. 2019), to build the initial reference. To ensure consistency, we mapped or relabeled some of the cell types to align with the reference and flow cytometry dataset. We maintained Plasmablasts as a separate cell type (rather than including them within B cells, despite their small population) because they formed a distinct cluster compared to other B cell subtypes in the reference data, as shown in Figure 16. We then constructed the cell reference matrix X^* by averaging the profiles of each cell type. For this reference, we retained 1343 intersecting genes shared between the sorted RNA-seq and bulk RNA-seq expression data.

Additionally, we incorporated another cell reference dataset derived from microarray technology into our analysis. We utilized LM22, the signature matrix from CIBERSORTx (Newman et al. 2015, 2019), which represents 22 distinct PBMC cell types characterized by 547 genes. From these, we selected 503 genes that were shared between the bulk RNA-seq expression data and LM22. Similarly to the other reference, we relabeled certain cells and then computed the average for each cell type.

A.4.2 Configurations of competing methods for benchmarking deconvolution tools on RNA-seq data

For benchmarking purposes, we provided BayesPrism and CIBERSORTx with the signature data containing original cell types in both data sets, i.e., sorted RNA-seq and microarray data. After the deconvolution step, we aggregated the proportions of the corresponding cell types, according to the mapping we applied in Section A.4.1, to ensure consistency with the flow cytometry data. This enabled us to calculate the quality scores involving cell abundances. For example, we

**Algorithm**	BayesPrism	CIBERSORTx(LM22)	CIBERSORTx (Harp)	BayesPrism (Harp)
Harp	0.300	0.161	0.025	0.264
**Hybrid deconvolution**				
CIBERSORTx (Harp)	0.925	0.835	–	0.908
BayesPrism (Harp)	0.543	0.361	0.092	–

**Algorithm**	BayesPrism	CIBERSORTx(LM22)	CIBERSORTx (Harp)	BayesPrism (Harp)
Harp	2.2×10^{-12}	1.4×10^{-15}	0.174	0.314
**Hybrid deconvolution**				
CIBERSORTx (Harp)	1.0×10^{-9}	1.7×10^{-12}	–	0.675
BayesPrism (Harp)	6.0×10^{-11}	6.3×10^{-14}	0.325	–

Table 7. Z-test statistics on Fisher's transformed cell type-wise, R_c, (top table) and sample-wise, R_s, (bottom table) correlation coefficients of Harp against other methods on deconvolution of RNA-seq gene expression data using a microarray-based reference data. The algorithm indicated by the table's row is tested for significance against the algorithm indicated by the column. Results are shown for the most representative run (i.e., one similar to the mean performance).

**Algorithm/ Metric**	**R**	**RMSD**	**mAD**	R_s	ρ
Harp	**0.931** ± 0.016	**0.090** ± 0.012	**0.068** ± 0.0073	**0.935** ± 0.014	0.907 ± 0.023
BayesPrism	0.753 ± 0.009	0.157 ± 0.003	0.120 ± 0.002	0.751 ± 0.009	**1.00** ± 1.28×10^{-7}
CIBERSORTx(LM22)	0.729 ± 0.165	0.165 ± 0.002	0.121 ± 0.003	0.728 ± 0.007	0.895 ± 0.002
**Hybrid deconvolution**					
BayesPrism (Harp)	0.913 ± 0.049	0.095 ± 0.023	0.073 ± 0.019	0.915 ± 0.051	**1.00** ± 1.28×10^{-7}
CIBERSORTx (Harp)	0.896 ± 0.057	0.108 ± 0.025	0.084 ± 0.021	0.902 ± 0.054	0.934 ± 0.026

**Algorithm/ Cell type**	**Monocytes**	**T cells**	**NK cells**	**B cells**
Harp	**0.712** ± 0.052	0.637 ± 0.080	0.615 ± 0.178	0.607 ± 0.071
BayesPrism	0.687 ± 0.062	**0.696** ± 0.079	0.682 ± 0.019	**0.691** ± 0.108
CIBERSORTx(LM22)	0.693 ± 0.044	0.685 ± 0.085	**0.715** ± 0.014	0.669 ± 0.091
**Hybrid deconvolution**				
BayesPrism (Harp)	0.710 ± 0.061	0.685 ± 0.089	0.634 ± 0.058	0.674 ± 0.115
CIBERSORTx (Harp)	0.660 ± 0.102	0.607 ± 0.108	0.451 ± 0.185	0.444 ± 0.183

Table 8. Evaluation of performance metrics for RNA-seq deconvolution using a microarray-based reference across five runs on 100 validation samples, for both the main and hybrid deconvolution benchmarks. Harp was trained on 20 training samples. Improvements in performance for each algorithm when using Harp's reference are shown in blue. The best performance in the main benchmark is highlighted in **bold**.

combined the predicted proportions of B cell subtypes and labeled the aggregated result as "B cells". In Figure 5, there is no correlation score for Plasmablasts from CIBERSORTx (LM22), because the reference does not include this cell type. Regarding the use of BayesPrism and CIBERSORTx (LM22) in our RNA-seq data benchmark, we mainly followed the procedures outlined in Section A.3.6. When providing LM22 to BayesPrism we set the parameter input.type = "GEP", as only one gene expression signature for each cell type was available. However, it is important to emphasize that BayesPrism is specifically designed to work with scRNA-seq expression data, whereas microarray data is not explicitly supported. For CIBERSORTx (RNA-seq), we enabled B-mode batch correction to deconvolute the bulk samples, using the sorted RNA-seq data as the source GEP (gene expression profile). Batch correction is highly recommended by the authors when the reference signature and bulk samples are measured on different platforms. Therefore, it was not strictly required in this case, as both data sets were measured through RNA sequencing. However, since enabling batch correction improved results for CIBERSORTx (RNA-seq), we decided to retain it. For both imputing cell type fractions and building a signature matrix, quantile normalization was disabled, as the input datasets were not derived from microarray technology. For CIBERSORTx (Harp), we enabled batch correction to be consistent with CIBERSORTx (RNA-seq), but did not select a GEP source in this case. Here, we emphasize that for quality performance regarding cell proportions, we excluded the Unidentified cells from the analysis. We did not include MuSic (Wang et al. 2019) in these benchmarks because it requires the entire single-cell library as input for the reference, which was not compatible with our reference data. To assess the quality of the reconstructed bulk expression profiles of the algorithms, we calculated the score for the predicted bulk samples for Harp as described in Section 2.2. For CIBERSORTx, this score is provided directly in the method's output, and for BayesPrism, we reconstructed bulk samples by summing the cell expression profiles for each sample, as provided in its output, and then calculated the correlations, ρ according to Section 2.2.

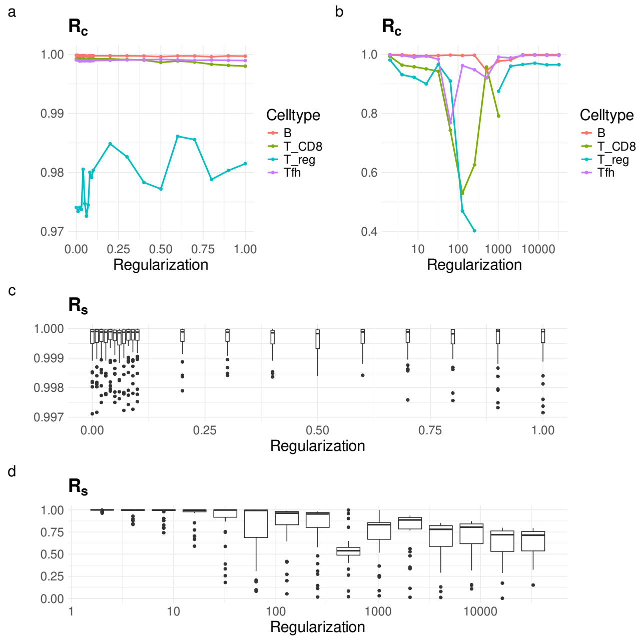

Figure. 15. Cell type-specific (a, b) and sample specific performance (c, d) of Harp for varying regularization parameters λ, separated in low (a, c), and large regularization (b, d) regimes.

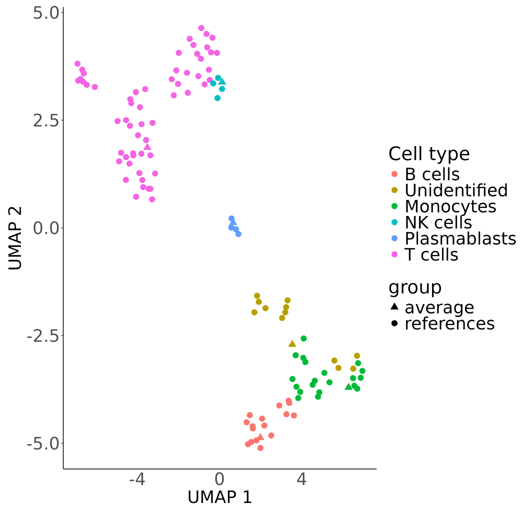

Figure. 16. Umap of sorted RNA-seq signature data (after mapping to the cell types corresponding to the flow cytometry data). The triangle marks the average profile of each cell type and each color represents a specific cell type.

A.4.3 Evaluation of reconstructed bulk expression profiles of RNA-seq data

With the aim of studying whether the source of cellular composition estimates/measurements or the adjustment of cell references have a greater influence on the accuracy of the reconstructed bulk gene expression profiles, we reconstructed two additional sets of bulk expression data. These reconstructions were similar to the previous ones (discussed in Section 3.2) but used cell abundances derived from Harp this time. In Table 9 and Table 10 the first and third row, with cell proportions labeled "Flow cytometry" were previously discussed in Figure 5 (g) and Figure 6 (g), respectively. Table 9 and 10 show that, in general, as expected, the adjustment of the reference profile (X in $Y = XC$) has a stronger influence on the quality of bulk expression predictions compared to the source of cell proportions. Comparing two rows with the same reference first and second or third and fourth, implies that the difference in the bulk predictions using different cellular composition matrices (C in $Y = XC$) is minimal, with only a very small advantage for Harp over the flow cytometry data. Moreover, a comparison of the two tables indicates that using a reference generated with a technology similar to the one the bulk expression data was generated with, in our case sorted RNA-seq reference, provides more accurate results.

Reference - cell proportions	Mean	SD
Harp - Flow cytometry	0.965	0.0181
Harp - Harp	0.976	0.014
Sorted RNA-seq - Flow cytometry	0.638	0.067
Sorted RNA-seq - Harp	0.641	0.069

Table 9. Comparison of reconstructed and observed bulk gene expression in 100 PBMC RNA-seq test samples. The mean and standard deviation of Pearson correlations (ρ) were calculated between reconstructed bulk expression profiles and observed RNA-seq data. The reconstructed bulk expression profiles were generated using the Harp reference and sorted RNA-seq data, with cell proportions from Harp and flow cytometry.

Reference - cell proportions	Mean	SD
Harp - Flow cytometry	0.881	0.0630
Harp - Harp	0.935	0.048
Lm22 - Flow cytometry	0.522	0.101
Lm22 - Harp	0.571	0.107

Table 10. Comparison of reconstructed and observed bulk gene expression in 100 PBMC RNA-seq test samples. The mean and standard deviation of Pearson correlations (ρ) were calculated between reconstructed bulk expression profiles and observed RNA-seq data. The reconstructed bulk expression profiles were generated using the reference from Harp and microarray data (LM22), with cell proportions from Harp and flow cytometry.

A.4.4 Hybrid deconvolution on bulk RNA-seq expression data using a sorted RNA-seq reference as Harp's anchor

Similarly to the simulation experiments, we provided the harmonized reference matrix learned by Harp to CIBERSORTx and BayesPrism, and compared the performance of this approach, to that achieved with their default LM22 reference (see supplemental Section A.4.2 for details). Figures 17 (h–j) and Table 3 show that Harp's reference improved the overall performance of both methods. Moreover, cell proportions within samples were better reconstructed when using the harmonized references (Figure 17 (l) and R_s in Table 3). For predicting cell type-specific abundance, R_c, see Figure 17 (k) and Table 3), CIBERSORTx benefited more from using Harp's reference than from constructing a custom reference from sorted RNA-seq data for predicting Monocytes, T cells and Plasmablasts populations, although CIBERSORTx (LM22) performed even better. Additionally, we observed that the Harp

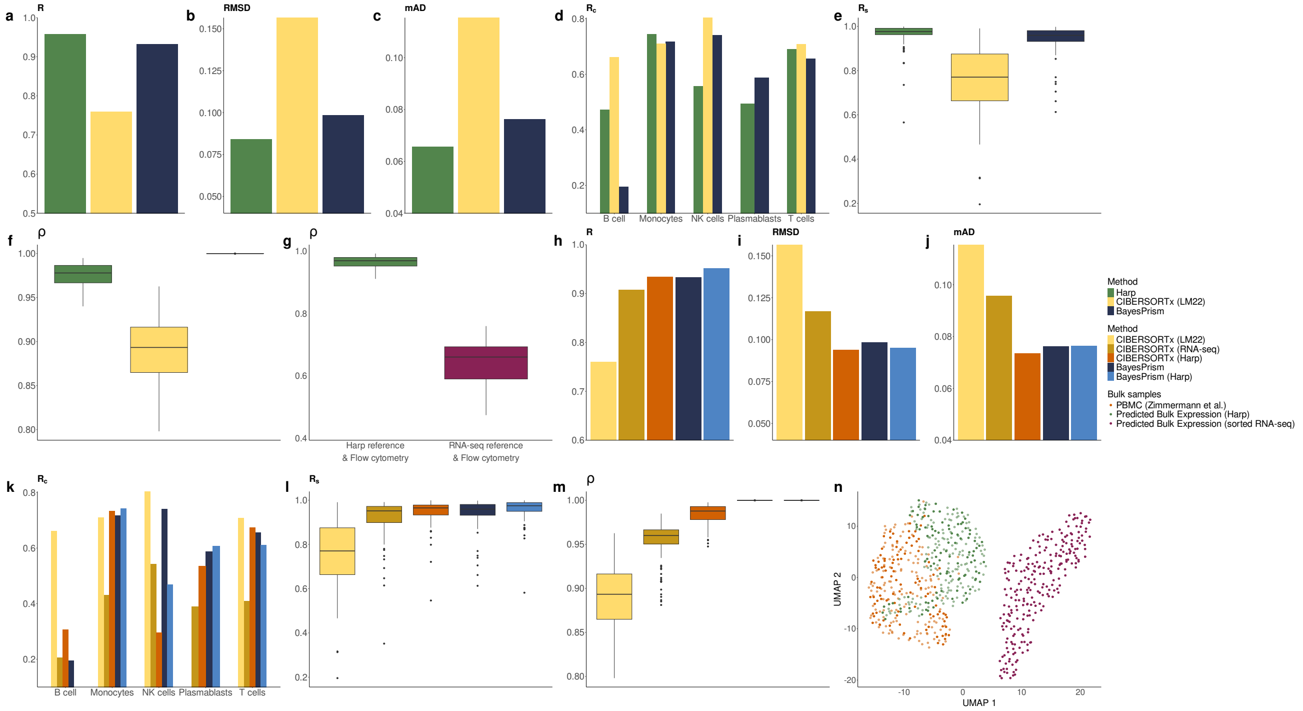

Figure. 17. Evaluation of performance metrics in the deconvolution of 100 PBMC RNA-seq test samples with a sorted RNA-seq reference. (a–f) represent the benchmark of deconvolution tools (Harp, CIBERSORTx (LM22), and BayesPrism). Plots (a–e) evaluate performance on the prediction of cell proportions, while plot (f) analyses the quality of the reconstructed bulk gene expression profiles. (g) shows box plots of Pearson correlations ρ between the reconstructed bulk gene expression profiles—generated using the experimental cellular compositions in conjunction with the Harp (green) and sorted RNA-seq derived (magenta) references, respectively—and the observed bulk RNA-seq data. (h–m) depict the hybrid deconvolution scenario of providing the harmonized reference matrix, X' (sorted RNA-seq reference as the anchor X^*), calculated by Harp to CIBERSORTx and BayesPrism, and these results are compared to those from the respective default methods. Plots (h–l) assess the accuracy of the estimated cell proportions, while plot (m) evaluates the quality of reconstructed bulk gene expression profiles. (n) is a UMAP of the predicted bulk gene expressions of 100 PBMC test samples (darker color shades) and 150 PBMC training samples (represented in lighter color shades), using the Harp (green) and sorted RNA-seq (magenta) reference, respectively, in conjunction with the cellular compositions derived from experimental data. This plot also includes observed bulk RNA-seq expression profiles (orange).

reference improved BayesPrism's prediction of Monocytes and plasmablast proportions, while it had a negative impact on the predictions for B cells, NK cells, and T cells proportions. Regarding the prediction of bulk gene expression (see Figure 17 (m) and ρ in Table 3), BayesPrism outperformed the other methods in both its default mode and when using the Harp reference. Moreover, with the harmonized Harp reference, CIBERSORTx showed improved performance compared to both CIBERSORTx (RNA-seq) and CIBERSORTx (LM22).

A.4.5 Hybrid Deconvolution on bulk RNA-seq expression data using a microarray-based reference as Harp's anchor

We again examined the effect of using harmonized references on the performance of competing tools. Figures 18 (h–k), Figure 18 (m) and Table 6 show that both CIBERSORTx and BayesPrism benefited from using Harp's reference. Notably, when CIBERSORTx was used with Harp's reference, no batch correction was performed when applying CIBERSORTx, yet its performance still improved. Regarding the prediction of cell type-specific proportions, Figure 18 (l) and R_s in Table 6 indicate that both methods achieved better performance using Harp's references for Monocytes and T cells, while for NK cells, and B cells performance was higher when using each method's default reference.

A.5 Harp's performance remained intact when using data from single microarray source

A.5.1 Benchmarking Harp against other deconvolution tools

As a second example using real data, we used 20 PBMC samples from (Newman et al. 2015), for which bulk gene expression was measured by microarray technology and cell proportions were measured via flow cytometry. For this dataset, we randomly split the samples into a training set consisting of 12 samples and a validation set of eight samples. We repeated the random data split five times and performed statistical analyses (see Section A.3.8, Table 11, and Table 12). The most representative run was selected to generate Figures 19 and 20 as well as Table 13. In these figures, we excluded cell type–specific performance results to avoid potential misinterpretation, as all algorithms exhibited high standard deviations. For the anchor X^* in Harp we utilized LM22 provided in (Newman et al. 2015) (for more details see Section A.5.4).

Here, we observed that, in general, Harp showed a strong performance, similar to its result discussed earlier. According to Figure 19 and Table 11, in overall correlation, R, Harp performed better than both BayesPrism and CIBERSORTx (LM22). However, BayesPrism achieved better RMSD and mAD in comparison to Harp and CIBERSORTx (LM22).

Algorithm/ Metric	R	RMSD	mAD	R_s	ρ
Harp	**0.884** ± 0.036	0.098 ± 0.020	0.070 ± 0.014	**0.854** ± 0.055	0.952 ± 0.010
BayesPrism	0.855 ± 0.030	**0.080** ± 0.009	**0.059** ± 0.006	0.818 ± 0.052	**1.00** ± 3.73 × 10^{-7}
CIBERSORTx (LM22)	0.744 ± 0.053	0.099 ± 0.008	0.072 ± 0.005	0.734 ± 0.041	0.842 ± 0.005
**Hybrid deconvolution**					
BayesPrism (Harp)	0.854 ± 0.034	0.122 ± 0.017	0.080 ± 0.012	0.816 ± 0.059	1.00 ± 3.73 × 10^{-7}
CIBERSORTx (Harp)	0.851 ± 0.039	0.092 ± 0.016	0.065 ± 0.013	0.826 ± 0.054	0.980 ± 0.003

Algorithm	Monocytes	Memory B cell	Naive B cell	$\gamma\delta$ T cell	CD4 T cell	CD8 T cell	NK
Harp	0.643 ± 0.266	0.276 ± 0.449	0.748 ± 0.119	0.422 ± 0.390	0.683 ± 0.205	0.754 ± 0.081	0.513 ± 0.421
BayesPrism	**0.701** ± 0.220	**0.406** ± 0.535	0.587 ± 0.156	**0.749** ± 0.120	**0.690** ± 0.118	0.682 ± 0.128	0.831 ± 0.148
CIBERSORTx (LM22)	0.676 ± 0.199	0.267 ± 0.506	**0.760** ± 0.106	0.333 ± 0.309	0.668 ± 0.172	**0.852** ± 0.055	**0.894** ± 0.064
**Hybrid deconvolution**							
BayesPrism (Harp)	0.684 ± 0.232	0.220 ± 0.431	0.680 ± 0.143	0.698 ± 0.099	0.689 ± 0.156	0.764 ± 0.079	0.619 ± 0.166
CIBERSORTx (Harp)	0.710 ± 0.257	0.291 ± 0.453	0.406 ± 0.372	0.695 ± 0.195	0.661 ± 0.080	0.399 ± 0.401	0.485 ± 0.511

Table 11. Evaluation of performance metrics for microarray deconvolution using a microarray-based reference across five runs on eight validation samples, for both the main and hybrid deconvolution benchmarks. Harp was trained on 12 training samples. Performance improvements for each algorithm when using Harp's reference are shown in blue. The best performance in the main benchmark is highlighted in **bold**.

Algorithm	BayesPrism	CIBERSORTx(LM22)	CIBERSORTx (Harp)	BayesPrism (Harp)
Harp	0.54	0.520	0.730	0.52
**Hybrid deconvolution**				
CIBERSORTx (Harp)	0.300	0.290	–	0.280
BayesPrism (Harp)	0.840	0.670	0.210	–

Algorithm	BayesPrism	CIBERSORTx(LM22)	CIBERSORTx (Harp)	BayesPrism (Harp)
Harp	0.11	0.006	0.180	0.130
**Hybrid deconvolution**				
CIBERSORTx (Harp)	0.390	0.055	–	0.420
BayesPrism (Harp)	0.500	0.030	0.350	–

Table 12. Z-test statistics on Fisher's transformed cell-type wise, R_c, (top table) and sample wise, R_s, (bottom table) correlation coefficients of Harp against the second best performing methods on microarray data using a microarray-based reference data. The algorithm indicated by table's row is tested for significance against the algorithm indicated by the column. Results are shown for the most representative run (i.e., one similar to the overall performance mean).

Regarding cell type-specific prediction across samples (R_c), as shown in Table 11, BayesPrism outperformed both methods in inferring the proportions of Monocytes, Memory B cell, Gamma delta T cell and CD4 T cells, while CIBERSORTx (LM22) performed the best for Naive B cells, CD8 T cell and NK cell.

Figure 19 (e) and R_s in Table 11 show that Harp outperformed its competitors in within-sample cell proportion predictions. Harp also achieved better performance than CIBERSORTx (LM22) in predicting bulk gene expression profiles, see Figure 19 (e) and ρ in Table 11.

A.5.2 Hybrid deconvolution

Similarly to Section 3.2 and 3.1.2, we studied the effect of the harmonaized Harp reference (using LM22 as anchor X^* as in the previous Section) on the performance of other tools in deconvolution of the eight PBMC bulk expression validation samples. In overall performance, Figures 20 and Table 11 show that CIBERSORTx benefited from the Harp reference, in contrast to BayesPrism. The Harp reference also influenced CIBERSORTx positively in predicting sample-specific cell proportions (see Figure 20 (d)). Table 11 represents that the performance of BayesPrism in predicting proportions of Naive B cells, CD8 T cells improved when using the Harp reference. However, the predictions for Memory B cells, CD4 T cells, Gamma delta T cell and NK cells were negatively affected when using the Harp reference.

On the other hand, CIBERSORTx showed better performance in predicting the populations of Monocytes, Memory B cells, and Gamma delta T cells when using Harp as a reference, whereas with LM22 performed better for the remaining cell types. In terms of bulk gene expression predictions, CIBERSORTx (Harp) outperformed CIBERSORTx (LM22) significantly, see Figure 20 (e) and ρ in Table 11. This indicates that Harp successfully compensated for inconsistencies between the cell reference and bulk expression data.

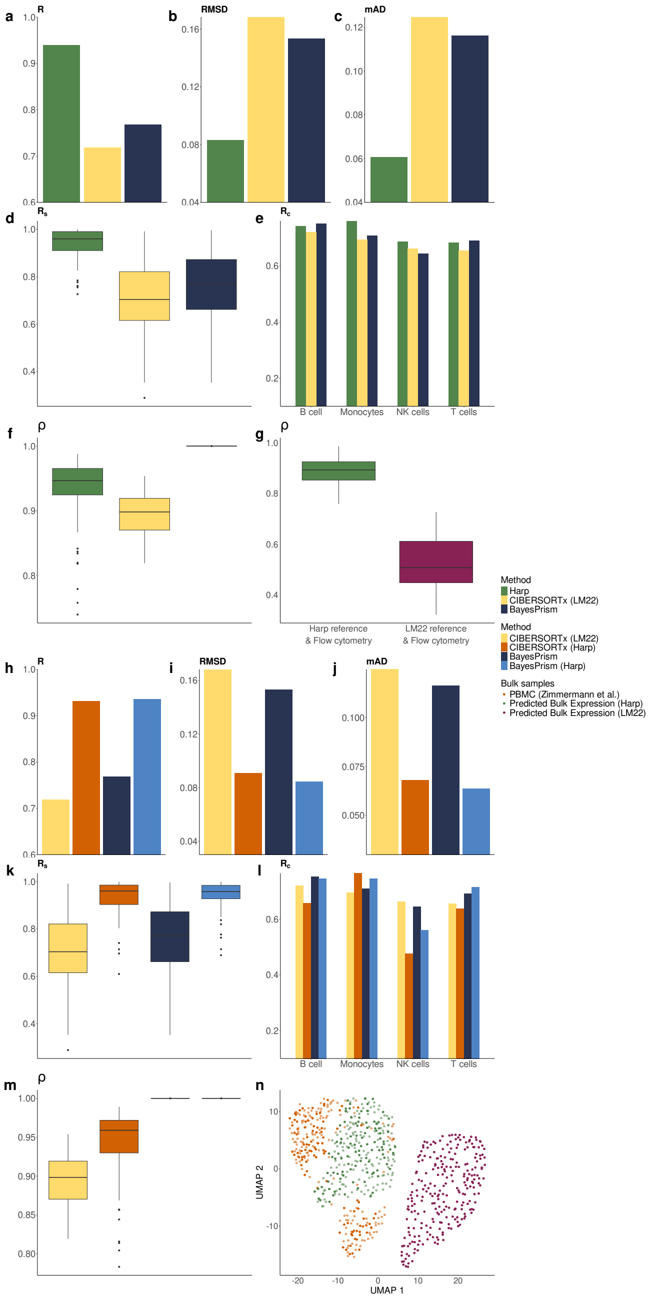

Figure. 18. Evaluation of performance metrics in the deconvolution of 100 PBMC RNA-seq test samples with a microarray-based reference (LM22). (a–f) represent the benchmark of various deconvolution tools (Harp, CIBERSORTx (LM22), BayesPrism). Plots (a–e) evaluate performance in predicting cell proportions, while Plot (f) analyses the quality of the reconstructed bulk gene expression profiles. (g) shows box plots of the Pearson correlation, ρ, between the reconstructed bulk gene expression profiles, using the flow cytometry-derived cell proportions, together with the Harp(green) and LM22 (magenta) references. (h–m) show the hybrid deconvolution scenario of providing the harmonized reference matrix, X' (microarray-based reference, LM22, as the anchor X^*), calculated by Harp to CIBERSORTx and BayesPrism, and the results are compared to those from their respective methods. Plots (h–l) evaluate performance in predicting cell proportions, while plot (m) analyses the quality of the reconstructed bulk gene expression predictions. (n) is a UMAP of the predicted bulk gene expressions of 100 PBMC test samples (darker color shades) and 150 PBMC training samples (represented in lighter color shades), using the Harp (green) and a microarray-based (magenta) reference (LM22), respectively, in conjunction with the cellular compositions derived from experimental data. This plot also includes observed bulk RNA-seq expression profiles (orange).

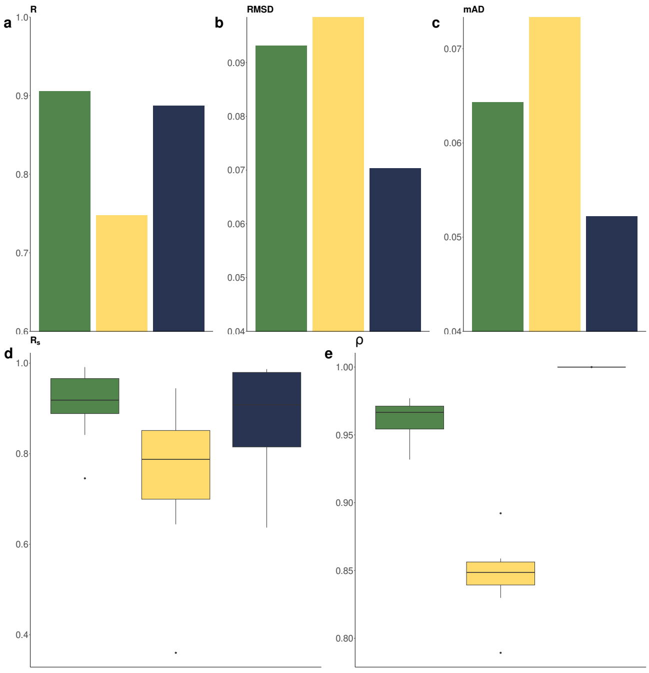

Figure. 19. Benchmark of various deconvolution tools (Harp, CIBERSORTx and BayesPrism) on eight PBMC microarray bulk expression validation samples with a microarray reference (LM22) using different quality scores. Plots (a–d) evaluate performance on the prediction of cell proportions, while plot (e) analyses the quality of bulk gene expression predictions.

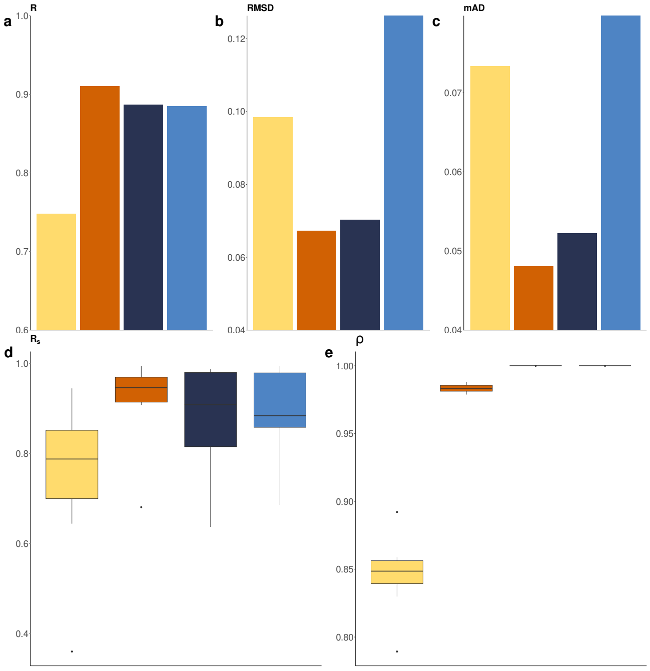

Figure. 20. Analysis of the impact of the Harp reference on CIBERSORTx and BayesPrism performance in deconvolution of eight PBMC microarray validation samples, comparing the results with the respective methods using the LM22 reference. Plots (a–d) evaluate performance on the prediction of cell type proportions, while plot (e) analyses the quality of the bulk gene expression predictions.

A.5.3 Evaluation of reconstructed bulk expression profiles of microarray data

Analogously to Section A.4.3 we studied the effects of different sources of obtained cell proportions and references in the reconstruction of bulk gene expression profiles. Table 13 shows that generally the effect of different references is stronger compared to cell proportions. Moreover, Harp explains the bulk expression data with a correlation of 0.96 , showing a major improvement in comparison to the LM22 reference.

In terms of reconstructing bulk expression data, using the Harp reference and LM22 along cell proportions from flow cytometry, the first and third row in Table 13 (as well as Figure 21a (a) , which provides another representation) show that the Harp reference improves the average correlations from about 0.39 to about 0.95 , demonstrating higher accuracy in explaining bulk samples compared to LM22. This difference is even more evident in the UMAP representation in Figure 21a (b). As can be seen in the plot, bulk gene expression profiles generated by the microarray-based reference formed a distinct cluster, while the bulk gene expression profiles using the Harp reference were more similar to the observed bulk expression data from the microarray measurement, and they formed one cluster together.

**Reference - cell proportions**	**Mean**	**SD**
Harp - Flow cytometry	0.948	0.018
Harp - Harp	0.961	0.016
Lm22 - Flow cytometry	0.389	0.044
Lm22 - Harp	0.388	0.048

Table 13. Comparison of reconstructed and observed bulk gene expression in eight PBMC RNA-seq test samples. The mean and standard deviation of Pearson correlations (ρ) were calculated between reconstructed bulk expression profiles and observed microarray data. The reconstructed bulk expression profiles were generated using the reference from Harp and microarray data (LM22), with cell proportions from Harp and flow cytometry.

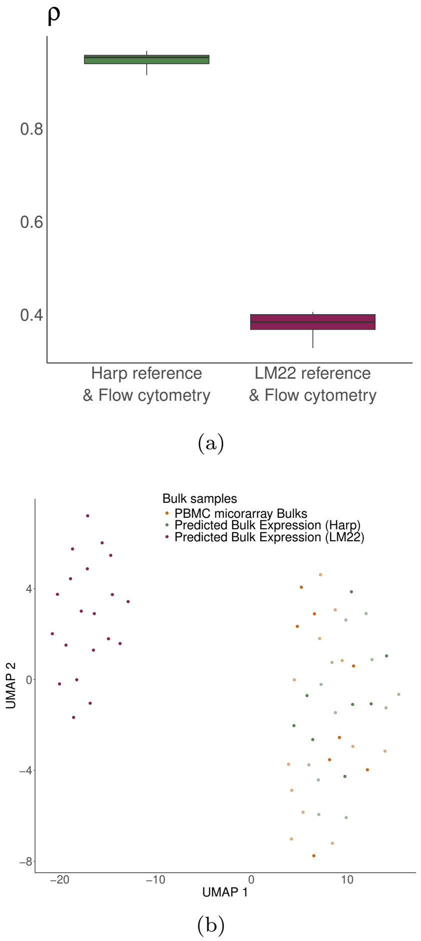

Figure. 21. (a) Comparison of the reconstructed bulk gene expression profiles. Box plots show Pearson correlations, ρ, between the reconstructed bulk gene expression for eight PBMC microarray samples, using Harp reference (green) and microarray-based reference (LM22) (magenta), and the observed microarray expression data. The cell proportions obtained from flow cytometry data were used for the reconstruction of the bulk samples. (b) is a UMAP of the predicted bulk gene expressions of 8 PBMC test samples (darker color shades) and 12 PBMC training samples (represented in lighter color shades), using the Harp (green) and a microarray-based (magenta) reference (LM22), respectively, in conjunction with the cellular compositions derived from experimental data. This plot also includes observed microarray expression profiles (orange).

A.5.4 Details of data processing of microarray data

The microarray bulk gene expression data included 20 PBMC samples from (Newman et al. 2015). For the anchor X^* in Harp we used the LM22 reference which is the standard CIBERSORTx signature (Newman et al. 2015). The bulk gene expressions were measured using the Illumina HumanHT-12 V4.0 expression beadchip platform, and the non-normalized data are available on NCBI under the GEO accession number GSE65133. Paired flow cytometry profiling was downloaded from the analyses of (Vallania et al. 2018).[4] The quantified cell populations include Monocytes, Memory B cells, Naive B cells, $\gamma\delta$ T cells, CD4 T cells, CD8 T cells, and NK cells. The proportions of all cell types summed up to 100%, indicating that the data was normalized before. In order to be able to use LM22 as anchor X^* in our method, we mapped or relabel some of the cell types to align with the flow cytomety data. Here, we did not include the Unidentified row and column in our input data. To utilize bulk expression data in Harp, we first mapped probe IDs to gene names using the R package illuminaHumanv4.db. Next, we calculated the average expression for probes with the same gene symbol within each sample to obtain per-gene expression values. Then quantile normalization was applied to the bulk samples. After splitting the data into training and test set, we trained Harp in its *Training* mode to estimate the Harp reference, we then used it in *Deconvolution* mode to deconvolute bulk samples in the test data set.

[4] The flow cytometry data is also available on NCBI, under the GEO accession number of GSE65133, under the 'Analyze with GEO2R' section (https://www.ncbi.nlm.nih.gov/geo/geo2r/?acc=GSE65133).

A.5.5 Configurations of methods for benchmarking deconvolution tools on microarray data

For tainting Harp in this scenario, due to the limited number of training samples, we performed 2-fold cross-validation instead of the default 5-fold. To impute cell fractions using CIBERSORTx (LM22), we provided CIBERSORTx with the gene expression data for all 20 samples, mapped to probe IDs. The data was not aggregated over similar gene names by

averaging, allowing CIBERSORTx to perform its own method to get the per-gene expression data. Quantile normalization was applied by CIBERSORT, and B-mode batch correction was also enabled, using the LM22 GEP source. Concerning CIBEROSRTx (Harp) and BayesPrism (Harp), we provided CIBERSORTx and BayesPrism with the Harp reference and the bulk samples as was in our test set. BayesPrism was provided with the anchor reference LM22, as described in Section A.4.2. We then calculated performance metrics only for the samples in the test sets.

For the reconstructed bulk data evaluation we followed the procedure described in Section A.4.2.
